# Multi‐Level Optical Physical Unclonable Function Based on Random Surface Scattering for Hierarchical Cryptographic Protocols

**DOI:** 10.1002/advs.202512317

**Published:** 2025-10-02

**Authors:** Jeong Jin Kim, Min Seong Kim, Gil Ju Lee

**Affiliations:** ^1^ School of Electrical and Electronics Engineering Pusan National University 2, Busandaehak‐ro 63 beon‐gil, Geumjeong‐gu Busan 46241 Republic of Korea

**Keywords:** hardware security, image encryption, laser speckle, multi‐level authentication, physical unclonable function

## Abstract

Optical physical unclonable functions (PUFs) have emerged as a promising cryptographic primitive for next‐generation security. However, to harmonize with various modern networks, conventional optical PUF is inadequate due to a rigid key space with a fixed specification. This study implements hierarchically controllable randomness sources for multi‐level key generation, exploiting speckle characteristics. By adjusting illumination diameter, the speckle size can be modulated, allowing the extraction of three‐level keys with various lengths: 64, 256, and 1,024 bits. Performance evaluation reveals that all levels are superb in uniformity, uniqueness, reproducibility, and randomness. Moreover, it is proposed two feasible applications. The first is a hierarchical authentication architecture that spans from Internet of Things (IoT) devices to sensitive information, balancing security and resources. The second is a multi‐stage image encryption algorithm, exhibiting holocryptic performance superior to conventional XOR‐based encryption. This versatile platform sets a new paradigm for optical PUF, establishing a foundation for robust and expandable next‐generation security.

## Graphical abstract

This study proposed physical unclonable function based multi‐level key space generation scheme. By exploiting speckle characteristic, a size‐controllable structure is achieved and that is suitable foundation for multiple key extraction. The flexible key space supports various specifications for advanced applications, such as hierarchical authentication framework or multi‐stage image encryption.

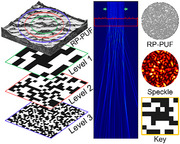

## 1 Introduction

The rapid growth in computational capabilities has revolutionized information processing across diverse fields.^[^ ^1^, ^2^ ^]^ This advancement presents potential security challenges^[^ ^3^ ^]^ as conventional security protocols become increasingly vulnerable to sophisticated attacks, including machine learning‐based approaches^[^ ^4^ ^]^ and emerging quantum computing threats.^[^ ^5^, ^6^ ^]^ In light of these threats, physical unclonable functions (PUFs) have emerged as promising primitives for next‐generation security architecture.^[^ ^7^, ^8^, ^9^ ^]^ Due to inherent randomness from physical or chemical fluctuation in the fabrication stage, PUFs provide hardware‐based encryption keys from physical storage.^[^ ^10^, ^11^, ^12^ ^]^ Among them, optical PUFs harnessing light‐matter interactions demonstrate potential prospects owing to a high degree of freedom in manufacturing and principles.^[^ ^13^ ^]^

Generally, strong interactions of light with specific particles or structures are widely used approaches to extract information from optical PUF medium. Various candidates have been explored in previous studies, including silk with fluorescent proteins,^[^ ^14^, ^15^ ^]^ fluorescence color,^[^ ^16^, ^17^, ^18^, ^19^, ^20^, ^21^ ^]^ Raman reporters,^[^ ^22^, ^23^ ^]^ plasmonic nanoparticles,^[^ ^24^ ^]^ and self‐focusing micro‐holes.^[^ ^25^ ^]^ These approaches yield stationary optical peak points for stable readout and expandability in encoding capacity using diverse precursors.^[^ ^26^ ^]^ An alternative embodiment of optical PUF exploits random surface morphology as an entropy source. Since the entropy is exposed externally, the readout of PUF is readily carried out using direct optical images^[^ ^27^, ^28^, ^29^, ^30^, ^31^ ^]^ or polarized images.^[^ ^32^, ^33^ ^]^ Owing to the solid structure, robust keys are ensured with fixed length corresponding to the dimension of superficial disorder.

However, responses of these approaches are strongly linked with a permanent structure fixed in the fabrication step, leading to a lack of flexibility in key space. This rigid key space faces a new challenge in the contemporary complexity of standalone devices.^[^ ^34^ ^]^ In efficient modern security frameworks, mutual communications or authentication protocols require various lengths of keys to balance resources and security.^[^ ^35^ ^]^ For example, the advanced encryption standards (AES) symmetric algorithm can be tailored to use 128, 192, or 256 bits of key depending on the required security level.^[^ ^36^, ^37^ ^]^ In addition to security, resources are another essential consideration. Conventional computing systems, unconstrained by resources, can introduce rigorous security algorithms using relatively long keys. On the other hand, lightweight algorithms with short encryption keys should be considered under resource‐constrained circumstances such as terminal sensor networks or wearable Internet of Things (IoT) devices.^[^ ^38^, ^39^ ^]^ Although key derivation can produce short keys from long keys, additional resource consumption and entropy loss are unavoidable.^[^ ^40^, ^41^ ^]^ In this regard, a capability to produce intrinsic hierarchical keys is beneficial to the PUF system to serve as a hub in an advanced security ecosystem.^[^ ^42^ ^]^ Among structure‐based optical PUFs, size‐configurable features are reported by modulating fabrication parameters.^[^ ^43^, ^44^, ^45^, ^46^, ^47^ ^]^ These platforms have a structural hierarchy in physical dimension, allowing multi‐length keys extraction, but still unchangeable during real‐time operation.

Herein, we propose a PUF‐based hierarchical cryptographic key generation scheme utilizing dynamically adjustable speckle topology. Speckle, representing a superposition of light passing through physical disorder, has proved excellent suitability as a deterministic source of randomness.^[^ ^48^, ^49^, ^50^, ^51^, ^52^, ^53^, ^54^, ^55^ ^]^ According to the well‐known physics of speckle described by Goodman,^[^ ^56^ ^]^ the speckle grain size can be modulated by adjusting geometrical parameters such as the illumination diameter or the detector distance. Of the two options, illumination diameter modulation can be readily achieved using a tunable aperture without significant system configuration change. This approach provides variable optical images by seamless transition across the morphological hierarchy, enabling the extraction of various‐length keys with a fixed PUF medium. As another consideration, multiple scattering in the PUF medium causes an undesirable expansion of the beam diameter at the rear surface,^[^ ^57^ ^]^ limiting the usable range of speckle sizes. To prevent this unwanted effect, we employed surface scattering with randomly distributed pits PUF (RP‐PUF) fabricated using a random wet etching process. As a result, we can obtain uncorrelated raw images categorized into three levels according to the speckle size. Speckles of various sizes have tailored spatial frequencies of bright and dark alternating points (i.e., potential source of “1” and “0” in binarized form). To extract binary encryption keys from various frequency speckle images, we designed an optimized process covering each level and successfully extracted the multi‐level key space consisting of 64, 256, and 1024‐bit keys. Utilizing this multi‐level key foundation, we propose a practical hierarchical authentication ranging from basic IoT devices to disposable key generation for confidentiality, achieved with a single PUF tag. Moreover, the hierarchical structure of the multi‐level key mediates superb image encryption performance, combined with the symmetric key algorithm. Utilized concepts to construct functional key space are described in Note S1 and Table S1 (Supporting Information).

## 2 Result and Discussion

### 2.1 Multi‐Level Key Space from Speckle Frequency Filtering

**Figure 1:**
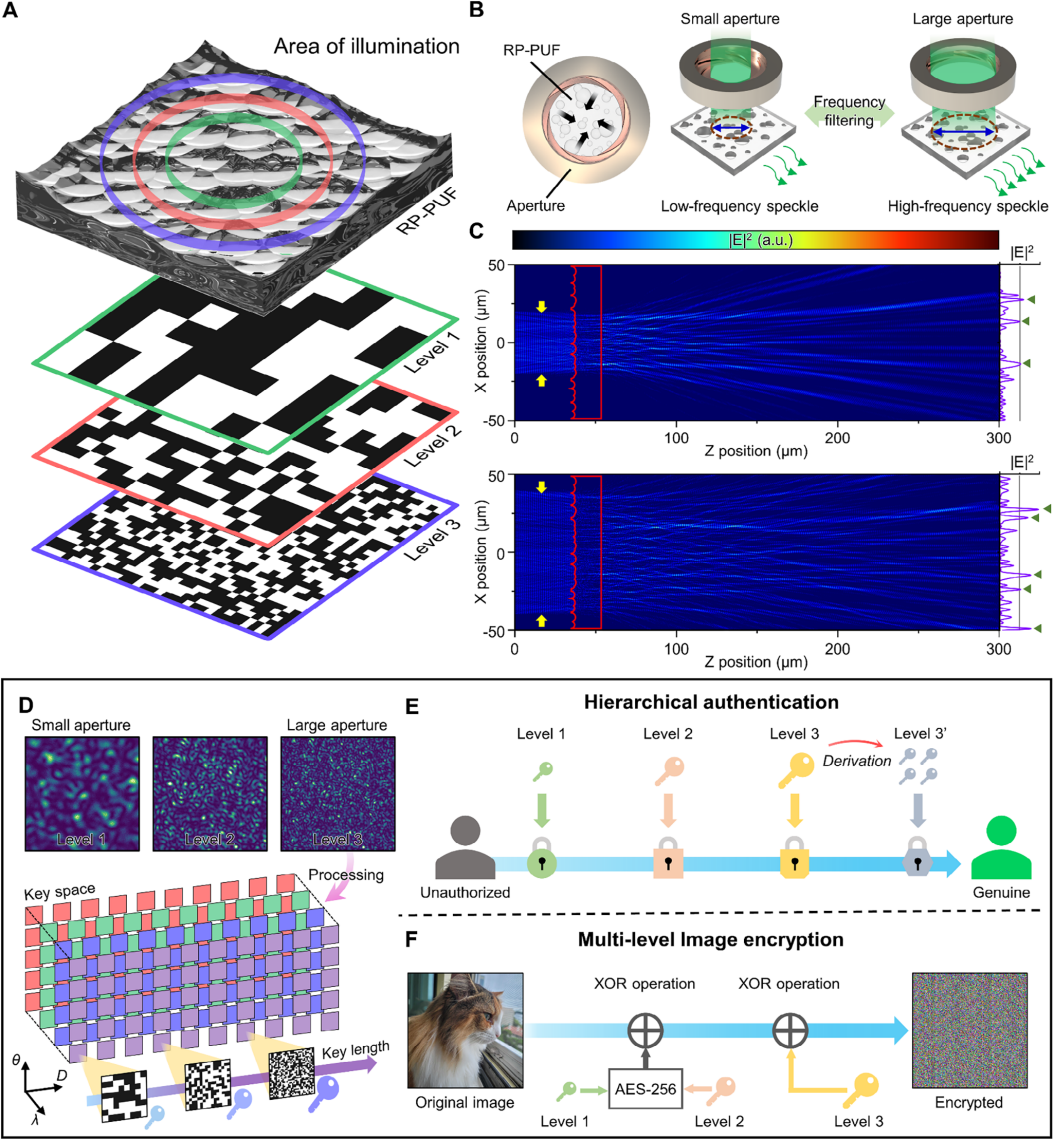
Multi‐level optical PUF system based on illumination diameter adjustment. A) Multi‐level key generation with various illumination areas. B) Challenge modulation through the aperture. By adjusting the aperture size, the width of the illuminated area changes, leading to a change in the spatial frequencies of speckle patterns. C) 2D simulation for illumination diameter indicated by yellow arrows. RP‐PUF is indicated by the red line. Narrow illumination generates sparse peaks, and wide illumination generates dense peaks. D) Speckle morphologies obtained with different illumination diameters and multi‐level key space. The key space comprises three levels featuring different key lengths. E) Hierarchical authentication with multi‐level keys. Each level can be used to satisfy the required security level and divided into disposable keys. F) Image encryption process with multi‐level keys. Images can be stored in an unrecognizable state.

When a disordered surface is illuminated by coherent light, a speckle pattern appears due to the superposition of randomly delayed phases. To induce unpredictable scattering for speckle generation, we utilize a RP‐PUF as a PUF medium (**Figure** **1**A). By illuminating the RP‐PUF at various scales, we can obtain multi‐level keys depending on the illumination area. Speckle characteristic mediates this relationship between the key size and the illumination area. The average speckle size depends on the illumination area and decisively affects the extractable key length. Therefore, constructing the multi‐level key space is contingent upon available speckle size steps.

The change of speckle morphology is readily achieved by adjustment of illumination area (i.e., aperture size), allowing seamless multi‐level key acquisition without PUF medium reconstruction (Figure 1B). Reduction of aperture size allows only pits of central region to engage speckle, leading to large and sparse speckle (i.e., low spatial frequency). In contrast, expansion of aperture size allows additional periphery pits into interference, producing small and dense speckle (i.e., high spatial frequency). Figure 1C displays the electric field intensity resulting from scattering behavior in the RP‐PUF. With the narrow launch width, only waves originating from central pits interfere, and sparse peaks are observed at the end of the domain (Figure 1C, top). With the wide launch width, more intricate scattering occurs, and significantly dense peaks are observed at the end of the domain (Figure 1C, bottom). The peak and valley are potential candidates of “1” and “0” after the binary key extraction process. Therefore, high spatial frequency at wide illumination implies a possibility of long key extraction with uniform distribution of bits, whereas low spatial frequency is suitable for short keys.

Figure 1D illustrates the overall key space of the RP‐PUF system. Under aperture adjustment, raw images with various speckle sizes are obtained. The images are classified into 3 levels according to the speckle size, and the optimized key extraction process for each level converts raw images into encryption keys. Accordingly, our system features a multi‐level key space consisting of 64, 256, and 1024‐bit encryption keys. Besides the aperture size (*D*), we verify the compatibility with other parameters contributing to speckle variation, such as wavelength (*λ*) and incident angle (*θ*). In our demonstration, 200 keys are available per RP‐PUF derived from a combination of ten aperture sizes, four wavelengths, and five incident angles.

Figure 1E depicts the hierarchical authentication process feasible with the proposed multi‐level key space of RP‐PUF. In this protocol, each step requires keys in various sizes or forms (e.g., larger for semi‐permanent passwords and smaller for disposable keys) according to the security level. However, PUFs with rigid key structures can only provide the keys in a fixed size, which is unsuitable for hierarchical authentication. Multi‐level key space resolves this problem by providing flexible key pools that can be utilized to match requirements in each hierarchical step. Moreover, division of a long key can provide disposable one‐time keys for additional stages as required (i.e., level 3′). Figure 1F exhibits a customized image encryption process utilizing the multi‐level key space of RP‐PUF. High‐resolution images require an extensive bitstream to be encrypted with a plain XOR operation.^[^ ^50^ ^]^ Even using a large bitstream, traces of the original images can be detected in the post‐encrypted image due to poor performance. Upon appropriate usage of the multi‐level key space, our customized image encryption algorithm can prevent vulnerabilities, ensuring a highly secure state.

### 2.2 Fabrication of RP‐PUF Suitable to Ensure Wide Modulation of Speckle Size

**Figure 2:**
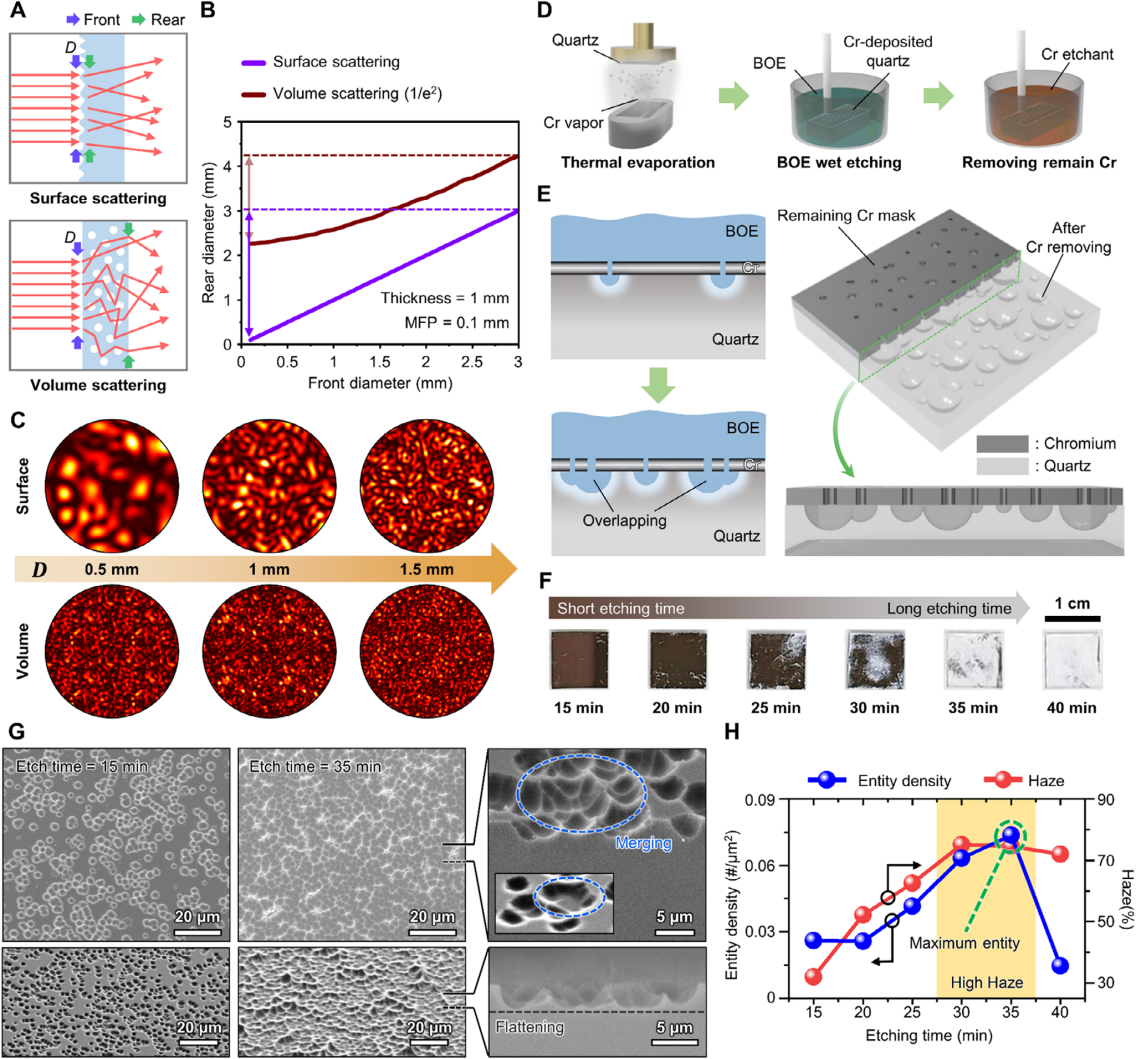
Analysis for the RP‐PUF and optimization. A) Influence of scattering type on the rear diameter. The front diameter is indicated by violet arrows, while the rear diameter is indicated by green arrows. Multiple scattering expands the rear diameter compared to surface scattering. B) Monte Carlo simulation result of surface scattering and volume scattering for illumination diameter expansion. Arrows indicate the modulation range for scattering type. C) Speckle simulation at narrow illumination region for scattering type. D) The overall fabrication of RP‐PUF using semiconductor processes. E) Detailed random pit generation as an entropy source of RP‐PUF. BOE stochastically generates isotropic pits on the quartz surface. As the etching time is longer, the pits overlap with each other. F) Photographs of RP‐PUF according to etching time G) SEM images and tilted view with etching times of 15 and 35 min, respectively. The right images show the merger of pits. H) Analysis of pits on the surface. Entities increase over time but decrease sharply in protracted duration.

Ensuring a sufficient range of speckle sizes is crucial to establish a hierarchical key space from raw speckles. This allows adaptation of extraction algorithms designed to generate keys of various lengths according to the speckle size. In free space geometry, the average diameter of speckle is proportional as follows:

(1) $$d\approx \frac{\lambda z}{D}$$

where *λ* represents wavelength, *z* represents the distance between the investigated surface and the observation plane, and *D* represents illumination diameter.^[^ ^56^ ^]^ Although both *z* and *D* affect speckle size, we only select *D* as a variable because the change of *z* can be more challenging in practical authentication systems.^[^ ^58^ ^]^ In surface scattering, *D* can be considered nearly equivalent to the actual illumination diameter. However, volume scattering introduces additional complexity (**Figure** **2**A). Multiple scattering provides more degrees of freedom to the light propagation channel in the medium, leading to expansion of actual illumination diameter (Figure S1, Supporting Information).

Monte Carlo simulation result of rear diameter expansion for front diameter is shown in Figure 2B. Detailed description regarding the volume scattering model is explained in Figure S2 (Supporting Information) and Experimental Section. Within the front diameter range of 0 to 3 mm, surface scattering exhibits a linear increase of rear diameter with front diameter, providing a modulation range of 3 mm. In contrast, the rear diameter modulation range is only ≈2 mm in volume scattering under the same conditions. Notably, the rear diameter of volume scattering starts from more than 2 mm based on the 1/e^2^ criterion, reducing the available rear diameter range. Although the influence of multiple scattering diminishes as the front diameter expands further, the volume scattering certainly has a disadvantage in terms of diameter modulation range. Based on the previous result, we conducted speckle simulation in the front diameter range from 0.5 to 1.5 mm (Figure 2C and Experimental Section). Speckles from surface scattering display a clear difference across diameters, whereas little change in morphology is observed in volume scattering due to early saturation. It reveals that surface scattering PUF is more suitable for introducing multi‐level key extraction subject to various speckle sizes.

For repeatable fabrication of surface scattering PUF with sufficient randomness, we adopted a stochastic wet etching process. Figure 2D illustrates the fabrication process of the RP‐PUF. The chromium (Cr) with 100 nm thickness is deposited on a quartz substrate as a partially protective mask for wet etching. Then, the sample is immersed in buffered oxide etchant (BOE) to make random pits on the Cr mask surface. After sufficient etching, the remaining Cr mask is removed by a Cr etchant, and an RP‐PUF tag is obtained. The optical microscopic images of samples before and after removing the Cr mask for etching time are shown in Figure S3 (Supporting Information). A random pit generation during wet etching is a critical point of PUF implementation (Figure 2E). Due to the instability of the Cr mask, pinholes are stochastically generated, and even partial delamination occurs. Then, the BOE infiltrates through the defects and etches the quartz in isotropic form. As hemispheric pits gradually expand, pits overlap each other and form a more complicated structure. To optimize etching time within BOE, samples are etched for 15, 20, 25, 30, 35, and 40 min, respectively (Figure 2F). In the case of 35 and 40 min, the residual Cr layer was hardly left. Figure 2G shows the top and tilted view of the scanning electron microscopy (SEM) images of representative samples with the etching time of 15 and 35 min. In the 15 min sample, each pit entity exhibits a distinct appearance. However, in the 35 min sample, some pits merge into a unified entity. In the cross‐sectional view with the protracted etching time, the hemispheric pits merge and become almost flat surfaces that are undesirable to trigger scattering. Every SEM image for all etching times is shown in Figure S4 (Supporting Information).

We analyzed detailed characteristics of randomly generated pits based on SEM images (Figure 2H). During the initial etching stages, distinguishable pit entities gradually increase. As the chrome mask weakens with longer etching time, the pit density progressively accelerates. However, it sharply decreases after 35 min because the quartz surface has no surficial capacity for additional pits, while already formed pits continuously merge. The haze (i.e., measure for light scattering) also increases to 30 min and then decreases thereafter due to flattening. Moreover, atomic force microscopy (AFM) reveals that the average etching depth was observed to be the deepest at 35 min. (Figures S5 and S6, Supporting Information). This indicates the most complex surface morphology at 35 min, which supports the high haze. Consequently, we selected 35 min as the optimal etching time to induce the most complex scattering. Detailed information about the fabrication of RP‐PUF is described in Experimental Section.

### 2.3 Demonstration of the RP‐PUF System for Variable Speckle Generation

**Figure 3:**
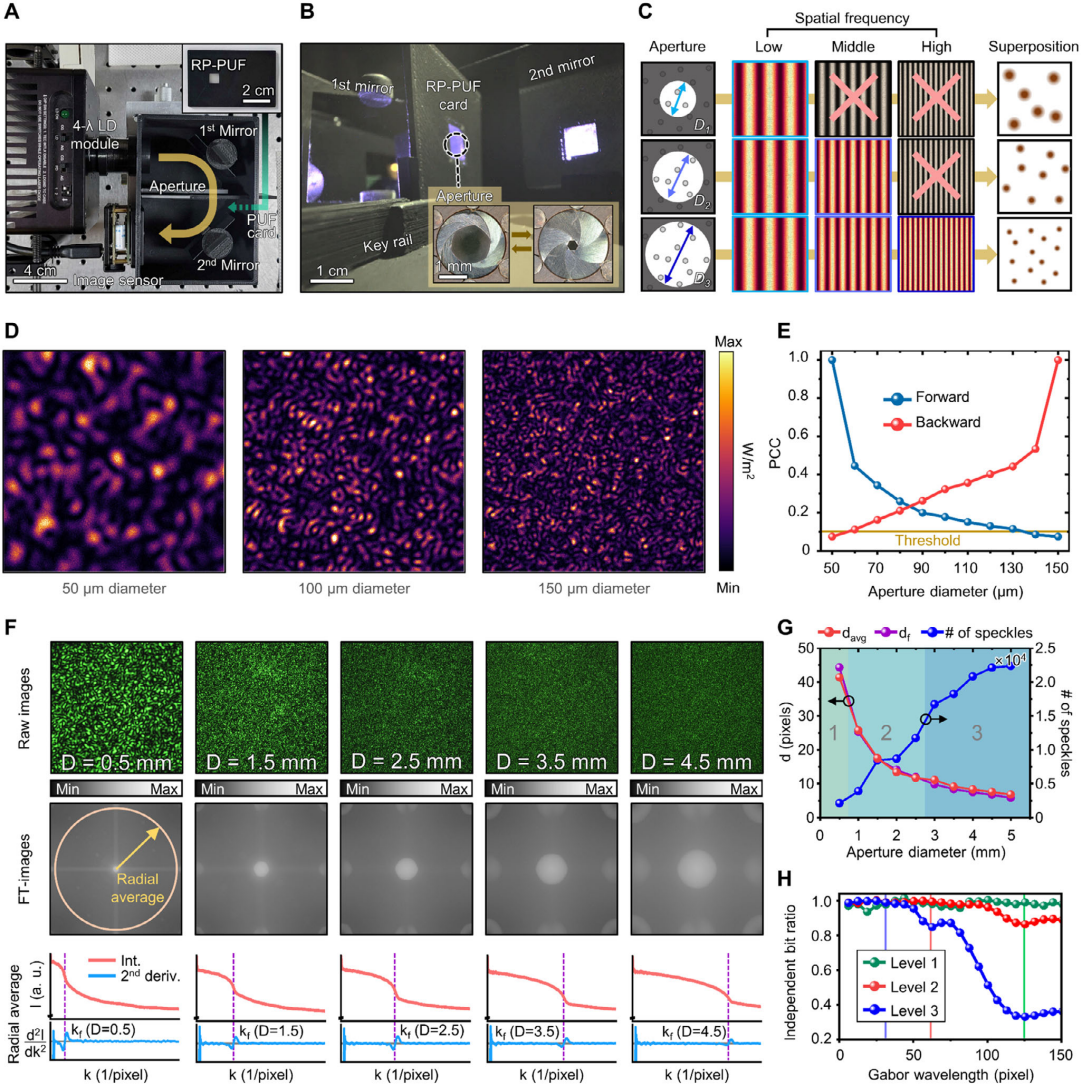
Demonstration of variable speckle generation with RP‐PUF. A) Top view of 3D‐printed authentication system. Light propagates in the direction of the yellow arrow. The inset shows a security card with RP‐PUF, and the green arrow indicates the inserting direction. B) The internal structure of the authentication system. The inset shows aperture adjustment. C) Interference frequency control based on aperture size adjustment. D) Monte Carlo simulation results with aperture size variation. E) PCC based on the aperture adjustment. The blue line is plotted forward while the red line is plotted backward. F) Spatial frequency analysis of speckle images with aperture adjustment; raw images (top), 2D‐Fourier spectra (middle), and radial average with 2nd derivative (bottom). G) Speckle size characteristics. As the aperture diameter increases, the number of peaks increases. Meanwhile, the mean radius d_avg_ decreases as well as a significant signal d_f_. H) Gabor wavelength optimization. Solid vertical lines indicate the selected Gabor wavelength for each level.

To acquire preliminary source speckles for the proposed multi‐level key space, we designed a customized 3D‐printed authentication system (**Figure** **3**A). The system consists of a laser diode (LD) module, two rotatable mirrors, the RP‐PUF card, an iris diaphragm, and an image sensor (Experimental Section). Utilizing the LD module and the rotatable mirror alongside the iris diaphragm, the key space is not only multi‐level but also significantly expanded (Figure S7, Supporting Information). The RP‐PUF is prepared into a security card for authentication convenience (Figure 3A; inset and Figure S8, Supporting Information). The internal space, including the inserted security card and two mirrors, is shown in Figure 3B. The iris diaphragm between the first mirror and the security card can dilate or constrict to adjust the illumination diameter (Figure 3B; inset). Figure 3C depicts spatial frequency filtering resulting from aperture size adjustment. The speckle pattern can be considered a superposition of interference patterns between tons of pit pairs. The spatial frequency of each interference fringe pattern is determined by the distance between two pits contributing to the interference. Therefore, the maximum spatial frequency included in speckles depends on the maximum distance between pits, adjusted by the aperture size. Under narrow illumination (i.e., *D_1_*), only close pit pairs in the exposed region can generate low spatial frequency patterns, leading to large and sparse speckle. As the aperture size increases (i.e., *D_2_* or *D_3_*), more distant pit pairs contribute to the speckle formation with a higher spatial frequency pattern.

Before the actual experiment, we conducted Monte Carlo simulations to prove distinguishability across variable speckles (Experimental Section and Figure S9, Supporting Information). Figure 3D presents the irradiance maps with the illumination diameter in 50, 100, and 150 µm, with a fixed 520 nm wavelength and normal incidence. A noticeable reduction in the speckle size is observed as the aperture size enlarges. Pearson's correlation coefficient (PCC) quantitatively reveals the uniqueness between seed images. The threshold is set at a PCC of 0.1 or less to confirm sufficient uncorrelation between two images for key generation. Figure 3E shows the PCCs calculated in the aperture diameter range from 50 to 150 µm. The blue line plot uses the 50 µm image as a reference for forward PCC, while the red line plot uses the 150 µm image as a reference for backward PCC. The PCC falls below 0.1 as the aperture diameter changes and key generation viability is validated. Simulation results regarding other factors are included in Figures S10 and S11 (Supporting Information), contributing to key space expansion such as wavelength and incident angle. The speckle changes with these parameters can be delineated based on overcoming the memory effect in spectral^[^ ^59^ ^]^ and spatial^[^ ^60^ ^]^ aspects.

The raw speckle images from actual experiment visualize the spatial frequency filtering for the aperture size (Figure 3F). To analyze speckle images in a frequency domain, we considered five speckle images (i.e., *D* = 0.5, 1.5, 2.5, 3.5, and 4.5 mm), as shown in Figure 3F; top row. Subsequently, 2D Fourier spectra are retrieved from the representative images (Figure 3F; middle row), described in Note S2 (Supporting Information). Each point on the shifted 2D‐Fourier spectra represents a specific spatial frequency in the original image. The magnitude of the frequency is the distance from the center (i.e., farther from the center means higher spatial frequency), while brightness means the intensity of the frequency. A larger aperture allows high‐spatial frequencies to pass, spreading the frequency distribution from center to periphery. The radial average of the 2D‐Fourier spectra elucidates the frequency filtering (Figure 3F; bottom row). As spatial frequency increases, the intensity (red line) rapidly decreases at a certain frequency for each illumination diameter. The evident inflection point (purple dashed line) is observed in the second derivative plot (blue line), excluding the initial sharp increase due to DC components and other minute fluctuations. The frequency at that point is the filtering frequency (i.e., *k_f_*) for the aperture size. Therefore, adjustment of aperture size can control the maximum significant spatial frequency. The 2D‐Fourier spectrum analyses for all aperture sizes are shown in Figure S12 (Supporting Information). Moreover, manipulation of wavelength and incident angle enables acquisition of independent raw images for seeding immense key space (Figure S13, Supporting Information).

To develop the speckle size controllability into a multi‐level key generation, we investigate speckle morphology in individual images (Figure 3G). As the aperture expands, the physical average radius of peaks (i.e., *d_avg_*, red line) decreases while the number of peaks (i.e., blue line) increases. Moreover, a minimum period of a significant spatial signal (i.e., violet line) is defined as follows:

(2) $$d_{f}=\frac{Image\;width}{k_{f}}$$

High similarity between *d_avg_* and *d_f_* represents that a smaller speckle is resulting from the contribution of more detailed spatial signals. Therefore, a wide and bright spectrum in the frequency domain means a large amount of spatial information. This appears in the spatial domain as a high density of peaks corresponding high frequency of solid bright and dark points, serving as a foundation to extract long keys. However, it is too cumbersome for implementation to design an individual key extraction process for every aperture size. Therefore, we classified the full range of aperture sizes into three levels based on speckle size for facilitation in practical applications (Figure S14, Supporting Information). Level 1 includes only 0.5 mm, level 2 ranges from 1 to 2.5 mm, and level 3 comprises 3 to 5 mm, indicated by the background number in Figure 3G. Wider aperture sizes can be used to further expand level 3 key space, but the Nyquist‐Shannon sampling limit should be considered to ensure acquisition of smaller speckles (Figure S15, Supporting Information). Finally, Gabor filtering is utilized to derive the hierarchy from speckles as a core mechanism of key extraction (Experimental Section and Figure S16A, Supporting Information). Among the parameters of the Gabor filtering, the wavelength of the kernel is crucial because it determines the dimension of the detectable structure in the image (Figure S16B, Supporting Information). If the Gabor wavelength matches the average speckle size, keys can aptly reflect the information of raw speckles.^[^ ^55^ ^]^ The adequacy of the key extraction process for each level can be evaluated with the independent bit ratio (IBR). IBR is an indicator of interrelationships between keys extracted from physical randomness, and an ideal value of unity means maximum independence^[^ ^61^ ^]^ as described in Note S3 (Supporting Information). As shown in Figure 3H, utilization of a large Gabor wavelength to small speckle (i.e., level 2 or level 3) results in a decrease of IBR. To accommodate entropy for key extraction, we select the level 1 Gabor wavelength and halve the Gabor wavelengths at each subsequent level (Figure S16C, Supporting Information) to maintain IBR near unit across all levels. The similarity between images at each level after Gabor filtering is found to be sufficiently low, confirming that the optimized filtering can generate independent sources for key generation (Figure S17, Supporting Information). Accordingly, the multi‐level key space consists of various keys of 64, 256, and 1024 bits according to the level.

### 2.4 Performance Evaluation of the Multi‐Level Key Space

**Figure 4:**
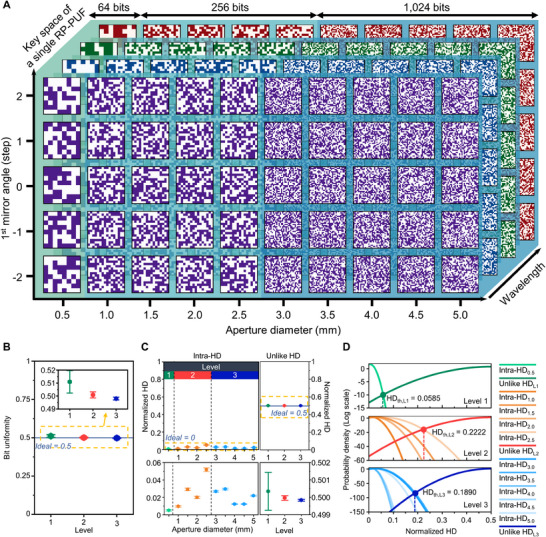
Key space and performance of RP‐PUF system. A) Total key space generated with a single RP‐PUF. 3D space has an inherent informational hierarchy. B) Bit uniformity at 3 levels distributed near the ideal value of 0.5. The inset shows a magnified view of the yellow dashed area. C) Intra‐HD for 10 aperture sizes and unlike HD for 3 levels. The measured metrics are close to the ideal value of 0 and 0.5, respectively. The bottom graphs present magnified views of the yellow dashed areas. D) Thresholds for the authentication scenario at each level. The threshold is determined at the intersection between intra‐HD and unlike HD.

Owing to the parametric divergence and hierarchical key extraction process, each RP‐PUF tag stores 200 keys (**Figure** **4**A). The key lengths are determined by the information volume at each level: 64‐bit in level 1, 256‐bit in level 2, and 1024‐bit in level 3. Moreover, the expansion across wavelength and angle further deepens the key space. To evaluate the performance of the RP‐PUF system, bit uniformity, reproducibility, and uniqueness are analyzed.^[^ ^62^ ^]^ The detailed information about performance metrics is described in Note S4 (Supporting Information). Bit uniformity reflects the bias of the extracted key. If the value is biased approaching 0 or 1, a higher risk of prediction arises due to low entropy. Evaluated bit uniformities of RP‐PUF at three levels are measured near 0.5, indicating unbiased (Figure 4B). Detailed bit uniformity distributions for each level are shown in Figure S18 (Supporting Information).

The reproducibility and uniqueness of the keys can be quantified using the normalized Hamming distance (HD) between keys. Intra‐HDs are retrieved from 300 self‐comparisons under the same repeated challenge, representing the robustness and reproducibility of the response. In the RP‐PUF system, the normalized intra‐HD mean values are close to zero across all aperture sizes, indicating the system can generate consistent responses under repeated measurements (Figure 4C; left). Intra‐HD distributions for the aperture size are provided in Figure S19 (Supporting Information). Furthermore, the availability of apertures smaller than 0.5 mm utilized for level 1 is investigated using intra‐HD, with detailed information described in Figure S20 (Supporting Information). Unlike HD, consisting of inter‐HD, unlike‐intra HD, and dual condition HD, signifies the uniqueness between keys generated from different challenges and samples (Figures S21 and S22, Supporting Information). To investigate a sufficient number of samples, we collected 1600 keys from 8 distinct RP‐PUF tags. Since unlike HD evaluates the independence between keys within the key space with a defined length, we calculated unlike HDs separately for each level. The normalized unlike HD mean values of the RP‐PUF system are distributed near 0.5 across all levels, denoting that the keys are unique and distinguishable from each other at all levels (Figure 4C; right). Unlike HD distributions for three levels are provided in Figure S23 (Supporting Information).

Based on the intra‐HD and the unlike HD characteristics, we can determine the threshold in scenarios where the RP‐PUF is introduced as an authentication root (Figure 4D). The distributions of normalized HD can be approximated by a normal distribution, where the threshold is determined at the intersection of intra‐HD and unlike HD. When multiple intra‐HDs compose the level, the rightmost value is selected to minimize the probability of false authentication (Figure S24, Supporting Information). Notably, the standard deviation of the unlike HD decreases as the key size lengthens (i.e., higher level) following a binomial distribution, significantly lowering the probability of false authentication. As a result, the false rate of multi‐level architecture drastically drops at higher levels. This feature is suitable for hierarchical authentication, allowing the allocation of keys considering the required security strength. Finally, to investigate the randomness of RP‐PUF more thoroughly, we conducted the NIST SP 800–22 randomness test suite (Table S2, Supporting Information). The bitstreams used for the test were constructed by concatenating all keys belonging to each level. The keys are derived from the combination of available parameters (i.e., aperture size, wavelength, and incident angle) and eight PUF tags. Initially, all bits from three levels were concatenated, and sufficient randomness was confirmed in a macroscopic sense. However, this combined collection consists of keys of various lengths, and the contribution from each level is highly unbalanced; the dominance of level 3 keys causes overlooking of bits from levels 1 or 2. Therefore, we performed the randomness test separately for each level, using only keys of identical length from one level. All test metrics yield p‐values higher than 0.01 and exceed the threshold proportion, indicating all levels retain sufficient randomness.

### 2.5 Versatility of Flexible Key Space and Hierarchical Authentication Platform

**Figure 5:**
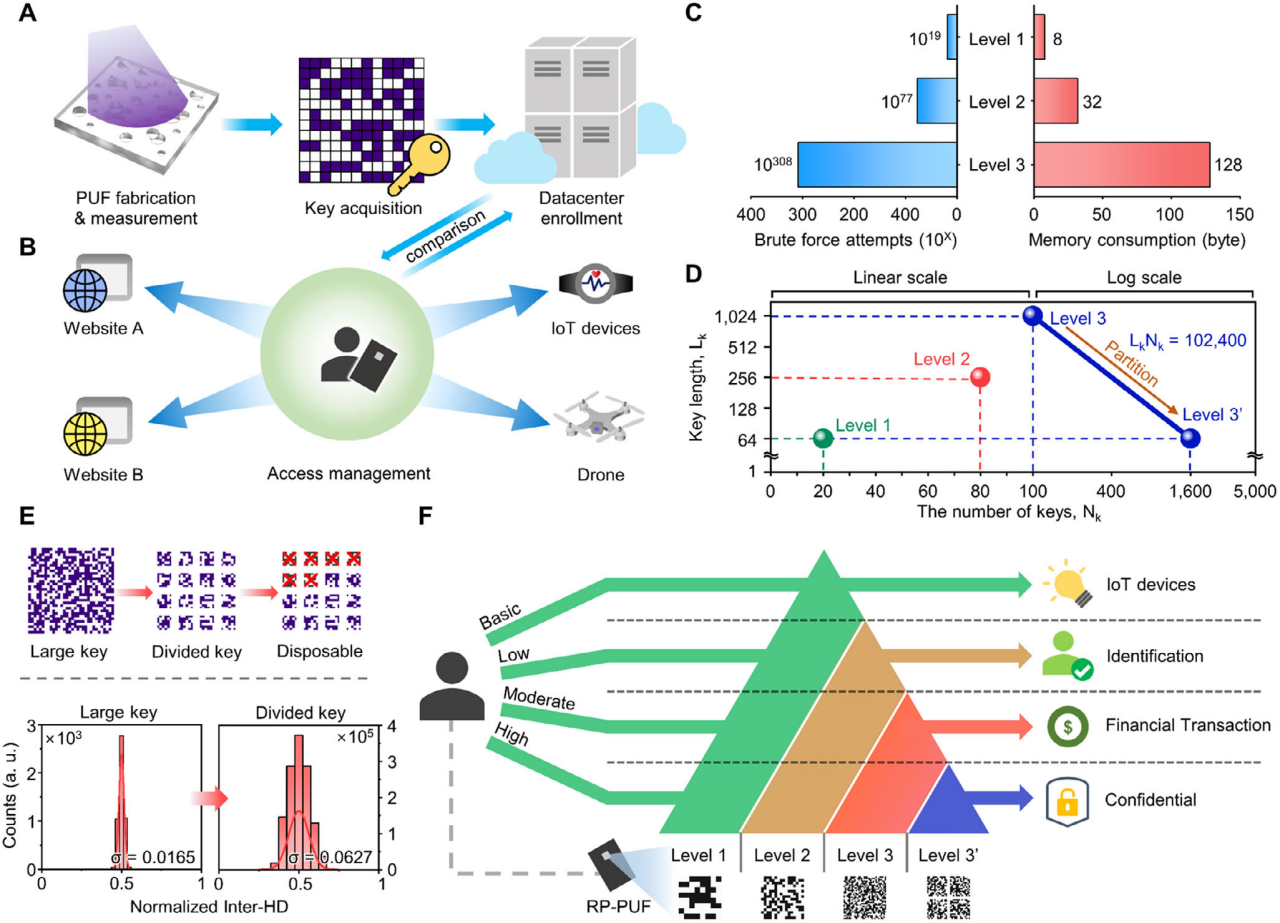
Layered authentication schematic based on hierarchical architecture. A) Schematic of the preliminary key enrollment step. After the transfer to a user, stored keys are called to compare with user‐generated keys. B) Key management for multiple communications in modern networks, with a single RP‐PUF card. C) Required brute force attempts and memory consumption for each level. D) Partition of encoding space for versatility. Large keys can be divided into sub‐keys according to the required specifications. The N_k_ from 0 to 100 is on a linear scale, while beyond is on a logarithmic scale. E) Disposable key generation for ephemeral protocols. Despite length reduction, the key performance is maintained as similar to that of level 1. F) The illustration of a hierarchical authentication platform tailored for various levels of security. Multiple levels can be simultaneously used to construct enhanced security.

The proposed RP‐PUF system features a hierarchical structure and large key space owing to the parametric modulation. These advantages can serve as a cryptographic platform to manage multiple identifications for diverse purposes and to implement hierarchical authentication. The basic authentication protocols based on optical PUF have been reported in several studies.^[^ ^7^, ^25^ ^]^ **Figure** **5**A depicts the preparatory steps for introducing RP‐PUF into authentication. After the manufacturing stage, keys are extracted by a trusted authenticator and stored in a database. Then, the PUF tag is transferred to the user. On demand of an authentication attempt, the user‐generated key is compared with the stored key for verification (Figure 5B). Owing to the extensive key space, multiple keys for various access can be stored in a single tag, serving as a “physical password manager”. In addition to the basic scenario, the multi‐level key space facilitates a balance of security strength and resources (Figure 5C). To quantitatively evaluate the trade‐off between security and resources, we consider required attempts for brute‐force attack and typical memory consumption. While lower‐level keys sacrifice some security by being relatively vulnerable to brute‐force attack, low memory consumption offers significant advantages under resource constraints. When communicating with environments requiring lightweight algorithms, such as IoT devices or drones, level 1 keys can be introduced. At the same time, higher‐level keys are available to communicate with conventional systems that are not limited by resource constraints, providing robust security.

In a more practical view, high‐level keys can be tailored by division process (Figure 5D). In our demonstration, the level 3 encoding space comprises 100 keys with 1024 bits within a single RP‐PUF tag. In this regime, total encoding space is given by the product of the number of keys N_k_ and key length L_k_, amounting to 102 400. This large encoding space can be reorganized into a small number of long keys or a large number of short keys. While long keys offer strong security, the rigid and fixed configuration constrains their versatility. By partitioning, the encoding space can be refined into short key form (i.e., 1600 keys with 64 bits). This form provides more divided keys of shorter length, suitable for disposable and ephemeral protocols such as one‐time passwords (OTPs) or session keys (Figure 5E; top). Although the strength of the key has relatively degraded due to length reduction as a trade‐off, it is still sufficiently secure as that of level 1 of the same length (Figure 5E; bottom). A more detailed scenario of the level 3 key division and disposal for OTP authentication is described in Figure S25 (Supporting Information). The comparison of utilizing an originally short key and splitting a long key in OTP authentication is discussed in Figure S26 (Supporting Information).

Figure 5F illustrates a schematic of layered security using the RP‐PUF system. Users can implement hierarchical security by stacking different security levels and applying them as needed within this platform. For basic scenarios for daily IoT devices, swift operation with minimal authentication (i.e., level 1) could be more beneficial. When requiring a higher security level for identification or sensitive financial purposes, higher levels (i.e., level 2 to 3) can be stacked to build a more rigorous procedure in addition to the basic algorithm using level 1. Finally, the most confidential data can be secured under disposable communication based on tailored keys (i.e., level 3′) from the key division. Additionally, the proposed key space can serve as a multilevel source for hash functions, providing bit arrays of the same length but with intrinsic security differentials (Figure S27, Supporting Information).

### 2.6 Hardware‐Based Image Encryption using the Classified Key Space

**Figure 6:**
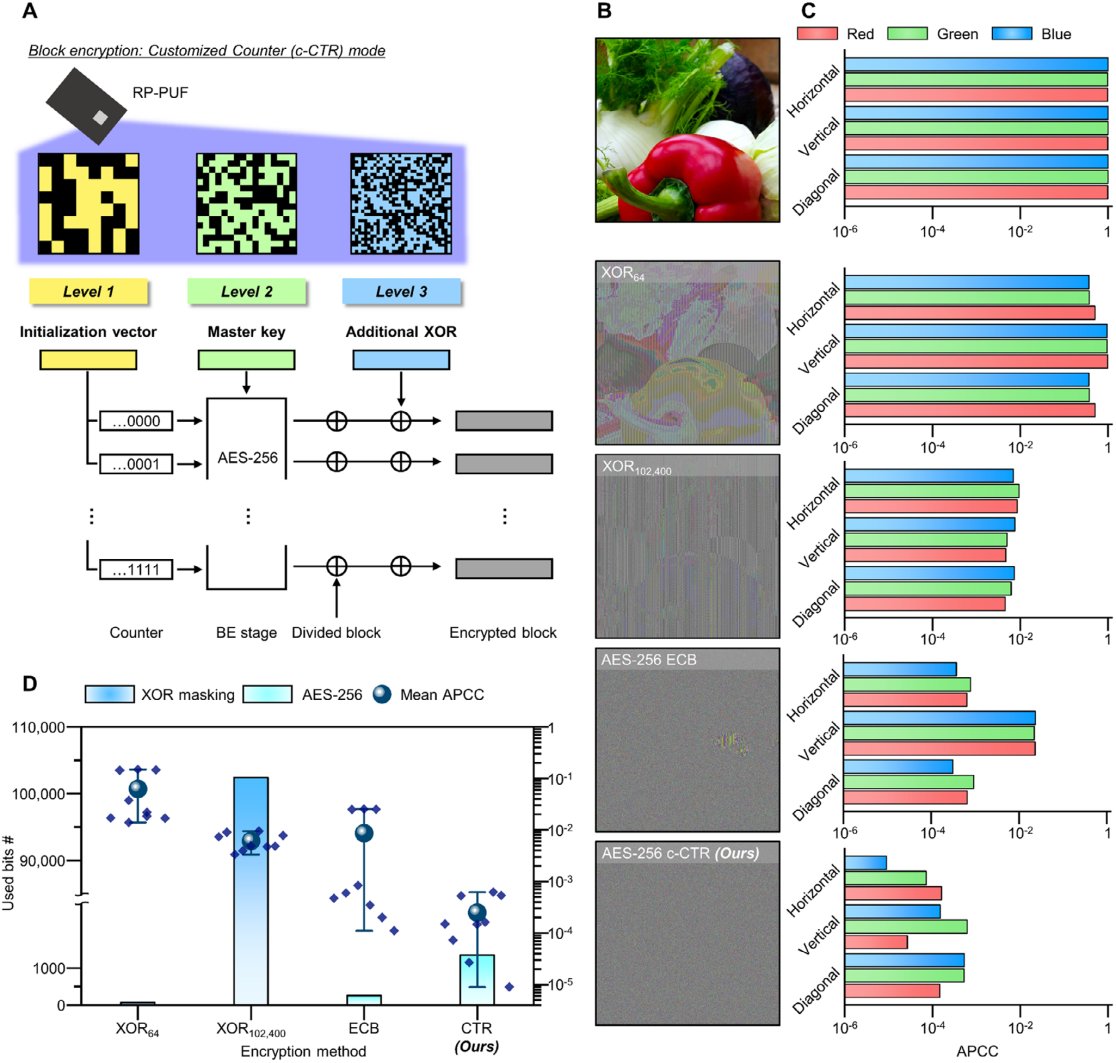
Image encryption demonstration with hierarchical key classification. A) Schematic of the proposed AES‐256 c‐CTR mode. Each encryption stage requires bits for the following three elements: initialization vector, master key, and additional XOR. Hierarchical levels of the RP‐PUF can match these three elements. B) Demonstration of image encryption candidates: XOR masked images (i.e., XOR_64_, XOR_102400_), ECB mode, and c‐CTR mode. C) APCC for the RGB channel and three directions. D) Bit usages and APCC distributions of image encryption protocols. The proposed c‐CTR shows significantly low APCC distribution and bits usage.

The hierarchical structure of the RP‐PUF can also be utilized in an encryption algorithm for high‐resolution image encryption. **Figure** **6**A illustrates a schematic of image encryption with an AES‐256 customized counter (c‐CTR) mode using the 3‐level classified keys from the RP‐PUF system. The c‐CTR mode operates with a counter consisting of two parts: an initialization vector and a block number, which increases by one per block. This counter is combined with a master key using AES‐256, producing independent encryption blocks. Subsequently, each divided plaintext block is processed by XOR with the corresponding encryption block, and then again with an additional XOR block. In this procedure, each level of RP‐PUF is employed to implement hardware‐based encryption; level 1 for initialization vector, level 2 for master key, and level 3 for additional XOR, respectively.

As a demonstration, we encrypt an original image using XOR masking (Figure S28A, Supporting Information), AES‐256 electronic code book (ECB, Figure S28B, Supporting Information) mode, and AES‐256 c‐CTR mode (Figure 6B and Experimental Section). Encrypted images for each protocol visually manifest the encryption performance. For XOR masking, the tiling for extracted bits is performed to match the image and key size. In simple XOR scenarios, the user can utilize only one level to compose a bitstream for XOR masking, therefore the representative unit tile length of 64 (i.e., one key from level 1) and 102 400 (i.e., all keys from level 3) bits are selected. XOR‐masked images have recognizable patterns or boundaries of the original image. On the other hand, AES‐256‐based algorithms encrypt images in an almost indiscernible state. However, recognizable boundaries remain in the ECB‐encrypted image while the c‐CTR‐encrypted image exhibits a holocryptic form.

For quantitative analysis of image protection protocols, the adjacent pixel correlation coefficients (APCC) for each encrypted image are shown in Figure 6C.^[^ ^63^ ^]^ The detailed APCC evaluation procedure is explained in Note S5 (Supporting Information). With the raw image, a high APCC is evaluated near 1 across all color channels and directions due to a continuous variation in pixel value. Conversely, encrypted images show extremely low APCC distribution due to the disappearance of correlations in adjacent pixels. In XOR_64_, APCC for all channels and directions represents a high value over 10^−1^. The XOR_102400_, which corresponds to the maximum unit tile size from non‐hierarchical usage of the RP‐PUF, indicates an APCC order of 10^−2^. With ECB mode, the vertical APCC of the image remains high and even higher than that of XOR_102400_. Our c‐CTR mode demonstrates a fully hidden feature and achieves the lowest APCC values compared to other image protection protocols.

Figure 6D shows the total bit usage and measured APCC for each encryption algorithm. The XOR masking with the short tile shows a significantly high APCC distribution, indicating inferior performance. Although the high APCC distribution can be alleviated by increasing the tile length, but still inefficient in terms of required bits (Figure S29, Supporting Information). By introducing AES‐256 into encryption algorithms, the APCC distribution can be notably reduced using fewer bits. ECB requires only a 256‐bit master key while providing APCC near that of XOR_102400_. However, ECB mode utilizes the same master keys for every divided block, leading to remaining tracks of the original images and high vertical APCC. The proposed c‐CTR protocol shows the lowest APCC distribution among encryption candidates. Due to multi‐level utilization, bit usage is slightly increased, but APCC distribution is meaningfully lower than sole XOR. APCC comparison for various images is illustrated in Figure S30 (Supporting Information); city, and candy. In summary, the multi‐level key space of the RP‐PUF can facilitate hierarchical hardware‐based encryption. This useful feature enables massive data encryption (e.g., sensors or medical data) with remarkable encryption performance.

## 3 Conclusion

In summary, we proposed an optical PUF system featuring a multi‐level key space using speckle. Speckle is superb physical entropy serving as a fingerprint of the optical PUF system, and illumination diameter adjustment can impart morphological hierarchy to speckles. To trigger the hierarchical divergence, we adopted a surface scattering medium fabricated with a stochastic wet etching process; RP‐PUF. We also organized the customized 3D printing authentication system comprising several parameters for key space expansion, such as wavelength, incident angle, and aperture size. Notably, aperture‐based spatial frequency limiting imparts an information hierarchy to speckle, enabling multi‐level key generation. The key space exhibited sufficient reliability in terms of bit uniformity, reproducibility, and uniqueness and passed the NIST randomness test suite across all levels. As a practical demonstration, we suggested the hierarchical authentication platforms applicable from IoT devices to confidential security. Moreover, a hierarchical image encryption algorithm based on multi‐level key space exhibits extraordinary APCC, compared with other encryption candidates.

Multi‐level key space proposed in this work presents two implications: 1) aperture size as a feasible parameter for key space expansion, and 2) flexible specifications from adjustable optical feature. In optical PUF using a speckle response channel, the available parameters in the system are directly linked with the key repertoire size (Figure S31, Supporting Information). In this context, we confirmed a new possibility of aperture size as a preferable key space expansion factor that can be combined with wavelength and incident angle, and furthermore, polarization or any adjustable material properties in future work. Moreover, variable‐length key space enables versatile applications such as hierarchical authentication or multi‐stage encryption. Such variable key specifications can also accommodate a wide range of devices requiring keys of different lengths. The hierarchical key space bridges the gap between optical PUF and modern communications, by supporting an extensive key space and multi‐level applications. We anticipate that this approach will be the cornerstone for a promising future of optical PUFs.

## 4 Experimental Section

### Monte Carlo Ray Tracing Simulation

It was used commercial software for geometric optical simulation, Ansys Zemax Opticstudio (Ansys, Inc., USA) to model speckle. Non‐sequential mode was employed to implement the scattering of rays. For the evaluation of rear diameter expansion, a scattering medium following Henyey‐greenstein phase function was considered, while the thickness and mean free path were assumed as 1 and 0.1 mm, respectively. A ray source with 1 × 10^6^ rays varying diameters was used, and the rear diameter was measured at the rear surface of the medium. For the speckle simulation, the same number of sources as the rear profile was placed and random phases were assigned. Each source launches 1 × 10^7^ rays to the detector located 100 mm away, and speckle profiles were obtained. For the observation of speckle divergence for system parameters such as aperture diameter, wavelength, and incident angle, the random pits distribution CAD was used. The scattering medium was reconstructed based on the mean and standard deviation of pit depths from AFM images. Lists for radii following the mean and standard deviation, and random X, Y positions were generated using the random number generator of MATLAB (MathWorks, Inc., USA). Finally, air spheres were distributed onto a glass sheet to imitate the RP‐PUF based on the generated lists. Ray sources used 1 × 10^10^ to 1.6 × 10^11^ rays depending on the aperture size to obtain reliable results with the Monte‐Carlo method. In all speckle simulations, the ray detector was operated with a coherent irradiant mode that collects information on the ray path length and calculates phase. Finally, all speckle images were reconstructed using OriginPro 2022 (OriginLab Co., USA).

### RP‐PUF Fabrication

A quartz diced into a 1 × 1 cm^2^ rectangular shape was cleaned with acetone in an ultrasonication bath for 30 min, followed by a rinse with isopropyl alcohol (IPA). The 100 nm Cr mask was deposited onto the quartz substrate (iTASCO, Republic of Korea) by thermal evaporation (KVT‐2000, Korea Vacuum Tech., Republic of Korea), with a deposition rate of 0.6 Å s^−1^. After forming the mask, the sample was immersed in the 6:1 BOE (Avantor, USA) to generate random micro pits on the surface. Subsequently, Cr etchant ETCR‐400 (Woo Jin Chem., Republic of Korea) removes the remaining Cr mask. The RP‐PUF was attached to a 3D‐printed card (M220, Moment Co., Ltd., Republic of Korea) with an optical adhesive named NOA61 (Norland Products, Inc., USA). Finally, the cards were cured for 5 min under ultraviolet light.

### Organization of the RP‐PUF Demonstration System

System housing was printed using a commercial 3D printer (M220, Moment Co., Ltd., Republic of Korea). Laser diodes with four wavelengths (L405P20/L450P1600MM/L520P50/L638P040, Thorlabs, Inc., Germany) were operated on a temperature‐controlled mount (LDM56/M, Thorlabs, Inc., Germany). Considering PCC for wavelength (Figure S10, Supporting Information) and that the laser diode mount LDM56/M only supports the package size of Ø 5.6 mm, four laser diodes are chosen. Mirrors were prepared by depositing Ag on a Si wafer and integrated with stepper motors (17HA2001‐03N, MOONS’, China) for rotation. The ion‐coupled plasma reactive ion etching (ICP‐RIE) cleaned the Si wafer with oxygen plasma for 10 s. The aperture size was controlled using a tunable iris diaphragm (D5S, Thorlabs, Inc., Germany). Speckle images were captured by a commercial CMOS image sensor (MT9J003, On Semiconductor Corp., USA). To fabricate Ag mirrors, sputter (DS‐2002, Korea, Pusan National University) and ICP‐RIE (SR‐2000, Korea, Pusan National University) are utilized. All components were securely assembled within a transition fit design.

### Key Extraction from Raw Speckle Image

The key extraction process was conducted using Python. First, captured raw speckle images were cropped into a 2700 × 2700 pixel size. These images were converted into grayscale and filtered using customized Gabor kernels. Gabor kernels for all levels have fixed parameters such as 135 ° of orientation, 1 of aspect ratio, and 0 of phase shift, while standard deviations were adjusted to 5, 10, and 20 to match the increase in Gabor wavelength with level. Subsequently, filtered images were resized to the key size based on the generated stripe images. Finally, an adaptive threshold was applied to binarize the images.

### XOR Masking

For all image encryption demonstrations, the pixel data from the original UHD image (3840 × 2160 × 8 × 3 bits) was rearranged to form a 1D bit sequence and separated into 128‐bit blocks. A discrete pixel value ranges from 0 to 255 (8 bits) for 3 color channels, and each pixel consists of 24 bits. For XOR masking, a unit tile bit extracted from an RP‐PUF is one‐dimensionally repeated to match the total size of the original image. This repeated bitstream was divided into three parts of the same length to be applied to three color channels: red, green, and blue. XOR operations were performed between divided bitstreams and each RGB channel of the original image. Finally, encrypted channels were merged to visualize the encrypted image.

### AES‐256 ECB Mode

The original image was divided into 128‐bit blocks and input to AES‐256 with the master key. All blocks were encrypted through the AES‐256 algorithm, using the same master key extracted from level 2 of the RP‐PUF. All encrypted blocks were gathered, and the collected bit array was resized to an original bit size of 3840 × 2160 × 8 × 3 bits to visualize the encrypted image.

### AES‐256 c‐CTR Mode

This image encryption protocol requires three elements: initialization vector, master key, and additional XOR. First, a counter block was composed of a 64‐bit increment and a 64‐bit initialization vector. AES‐256 receives the counter block and a 256‐bit master key as input. Then, the independent bitstreams as output were generated due to the counter increment. Each bitstream was XOR‐operated with the corresponding divided plaintext block. Finally, a 128‐bit additional XOR operation was conducted, and all encrypted blocks were concatenated. The collected bit array was resized to an original bit size of 3840 × 2160 × 8 × 3 bits for visualization. The RP‐PUF keys from level 1 were assigned to the upper 64 bits of the initialization vector, level 2 corresponds to the 256 bits of the master key of AES‐256, and level 3 was used for 8 divided blocks of additional XOR operation.

## Conflict of Interest

The authors declare no conflict of interest.

## Author Contributions

J.J.K. and M.S.K. contributed equally to this work. J.J.K. conceptualized and demonstrated the system by conducting experiments and analysis. M.S.K. fabricated and investigated the properties of the sample, and conducted image encryption algorithms. G.J.L. supervised the entire study.

## Supporting information



Supporting Information

## Data Availability

The data that support the findings of this study are available from the corresponding author upon reasonable request.
