# Supplementary material for: Multi‐Level Optical Physical Unclonable Function Based on Random Surface Scattering for Hierarchical Cryptographic Protocols
Source: Adv Sci (Weinh). 2025 Oct 2;12(46):e12317. doi: 10.1002/advs.202512317 (PMC12697780; doi:10.1002/advs.202512317)
Supplement: Supplementary file 1 — Supporting Information [file ADVS-12-e12317-s001.docx]

**Supporting Information**

**Multi-level Optical Physical Unclonable Function based on Random Surface Scattering for Hierarchical Cryptographic Protocols**

Jeong Jin Kim^1†^, Min Seong Kim^1†^ and Gil Ju Lee^1*^

^1^School of Electrical and Electronics Engineering, Pusan National University, 2, Busandaehak-ro 63 beon-gil, Geumjeong-gu, Busan, Republic of Korea, 46241.

*Correspondence to: gjlee0414@pusan.ac.kr

*†These authors contributed equally to this work*

**Supporting Notes**

**Supporting Note 1. Notable features for functional key space in optical PUF**

Hierarchical structures are achieved by adjusting fabrication parameters such as humidity or material proportion in self-assembly fabrication. Microstructures with various structural dimensions can serve various key lengths when coupled with an appropriate key extraction process optimized to extract features from the structure.

Multi-level has various meanings depending on the context of research. Multilevel PUFs commonly indicate systems using multiple response channels to expand encoding space. In another case of a multi-level optical PUF with a different meaning, even when using a single response channel, keys obtained by probing the PUF under variously modulated states are joined to increase effective key length. Furthermore, there is a novel research that demonstrates a hierarchical authentication using multi-level cryptographic keys. The multi-level is widely used to indicate multilayer structures from parallel response channels, externally modulated responses to hierarchical key shapes.

The concept of reconfigurability is defined as the capability to deliberately change its response to the same input challenge. In a narrow sense, this concept refers to an irreversible transition to a completely different PUF medium through an external physical process, such as melting and re-solidification. Subsequently, reconfigurability has evolved to include reversible mechanisms for modifying the medium to expand the key space.

In this paper, we implement the aforementioned features using the speckle characteristic. Speckles vary with aperture modulation, producing a multi-level response. This variation is accompanied by speckle size change, enabling a hierarchical topology for generating keys of various lengths. The key length specifications are defined at three levels, forming multi-level key space for advanced applications. Here, the term multi-level refers to multiple key specifications in this study. Finally, aperture size also determines the specific region probed by the challenge. Modulating the effective medium by including or excluding scattering centers at the boundary is equivalent to altering the PUF medium. Relevant literature regarding these concepts is summarized in **Table S1**.

**Supporting Note 2. Image Fourier transform for spatial frequency analysis**

To compare the filtered spatial frequency between images with various aperture sizes, the original images in the spatial domain should be converted into the spatial frequency domain. A 2D discrete Fourier transform expressed in **Equation S1** converts representative speckle images to a set of discrete sinusoidal spatial waves in complex number form.

$$\begin{aligned} F\left( u,v \right)=\frac{1}{WH}\sum_{x=0}^{W-1} \sum_{y=0}^{H-1} f\left( x,y \right)e^{-i2\pi\left( \frac{ux}{W}+\frac{vy}{H} \right)}=R\left( u,v \right)+iI(u,v)\#\left( 1 \right) \end{aligned}$$

where W and H denote the width and the height of the raw image, x and y represent the coordinates in the spatial domain, and u and v represent the coordinates in the spatial frequency domain.

The low-frequency components are positioned at the image corner with relatively much larger values than the high-frequency components. Therefore, the frequency set is shifted and converted to a logarithmic scale to facilitate visualization as expressed in **Equation S2**.

$$\begin{aligned} G\left( u,v \right)=\log(\left| F\left( u-\frac{W}{2},v-\frac{H}{2} \right) \right|)\#\left( 2 \right) \end{aligned}$$

As expressed in **Equation S3**, the complex number's magnitude denotes the point's intensity with a specific spatial coordinate in the spatial frequency domain.

$$\begin{aligned} \left| G\left( u,v \right) \right|=\left[ R^{2}\left( u,v \right)+I^{2}\left( u,v \right) \right]^{1/2}\#\left( 3 \right) \end{aligned}$$

We assumed the intensity maps have an isotropic profile. Finally, radial averages were calculated along the radial direction, and the filtering frequency was determined.

**Supporting Note 3. Encoding capacity of PUF**

The encoding capacity of PUF is expressed as *c^s^*, where *c* represents the number of possible output states and *s* represents the key length. Because our multi-level key system produces binary encryption keys, c is 2 and s is 64, 256, and 1,024 for each level.

However, the PUF system should introduce practical encoding capacity considering independence between bits evaluated with the degree of freedom (DoF) expressed as follows

$$\begin{aligned} DoF= \frac{\mu\left( 1-\mu\right)}{\sigma^{2}}\#\left( 4 \right) \end{aligned}$$

where *μ* is the mean of normalized inter-hamming distance (HD) and *σ* is the standard deviation. The practical encoding capacity is expressed as *c*^DoF^. Generally, the DoF is smaller than *s*. The independent bit ratio is defined as follows

$$\begin{aligned} Independent bit ratio= \frac{\mathrm{DoF}}{s}\#\left( 5 \right) \end{aligned}$$

The better the PUF system, including key extraction process, accurately reflects physical randomness and provides an independent key space, the closer DoF approaches *s* leading to the independent bit ratio becoming 1. The information about HD is described in **Note S3**.

**Supporting Note 4. Metrics for PUF performance evaluation**

Bit uniformity derives from the value of Hamming weight divided by the entire key size, followed by **Equation S6**, where *l* is the bit length and *B_i_* is the digit number in the whole bitstream *B*.

$$\begin{aligned} Bit Uniformity=\frac{1}{l}\sum_{i=1}^{l} B_{i}\#\left( 6 \right) \end{aligned}$$

HD is the number of different bits between two sequences of bits. To determine the HD of two sequences (*e.g.,* *B_x_* and *B_y_*), the XOR operation is applied to the corresponding bits at each position *i* (*e.g.,* *B_xi_* and *B_yi_*). The result is then normalized by dividing the HD by the bit length *l*. **Equation S7** expresses the normalized HD (NHD).

$$\begin{aligned} \mathrm{NHD}\left( B_{x} , B_{y} \right)=\frac{1}{l}\sum_{i=1}^{l} \left( B_{xi}\bigoplus B_{yi} \right)\#\left( 7 \right) \end{aligned}$$

The PUF system should be robust against external noisy environments. Therefore, the same PUF tag should produce the same final bit sequence under the same challenge input. **Equation S8** presents normalized intra-device HD (intra-HD) signifying the reproducibility of the PUF, where *B_x_^0^* is the original bit sequence and *B_x_^k^* is the *k*_th_ repeatedly extracted bit sequence under fixed conditions.

$$\begin{aligned} IntraHD=\left\{ \mathrm{NHD}\left( B_{x}^{0}, B_{x}^{k} \right) \right| k=1, 2, 3, \ldots\}\#\left( 8 \right) \end{aligned}$$

On the other hand, bit sequences generated by different PUF tags should be unrelated. The HDs between different tags, referred to as the normalized inter-device HD (inter-HD), imply the uniqueness of sequences. Inter-HD is estimated by **Equation S9**, where *m* and *n* denote the index of a set of bit sequences extracted from various PUF tags and challenges.

$\begin{aligned} InterHD=\{NHD\left( B_{m}, B_{n} \right) | \forall m<n\}\#\left( 9 \right) \end{aligned}$

**Supporting Note 5. Adjacent Pixel Correlation Coefficient (APCC) measurement**

The plain image maintains the structural continuity except for the boundaries of objects. The pixel intensity gradient is not significantly high for the entire region. In contrast, the encrypted images require minimal adjacent pixel correlation to increase the pixel value gradient with the cryptographic algorithm intentionally. As defining the pixels as for one color channel for C_xy_, where C is the color channel, x is the vertical position, and y is the horizontal position of the image. The original image is split into three color channels, red, green, and blue. Each channel is converted into a one-dimensional bit array to investigate the directional correlation. Then, Pearson’s correlation coefficient (PCC) measurement is performed in vertical, horizontal, and diagonal directions, and repeated for each color channel. Vertical pixel correlation is defined as a PCC between the {C11, C12, C13, …} and {C21, C22, C23, …}. Horizontal pixel correlation is defined as a PCC between the {C11, C12, C13, …} and {C12, C13, C14, …}. Diagonal pixel correlation is defined as a PCC between the {C11, C12, C13, …} and {C22, C23, C24, …}.

**
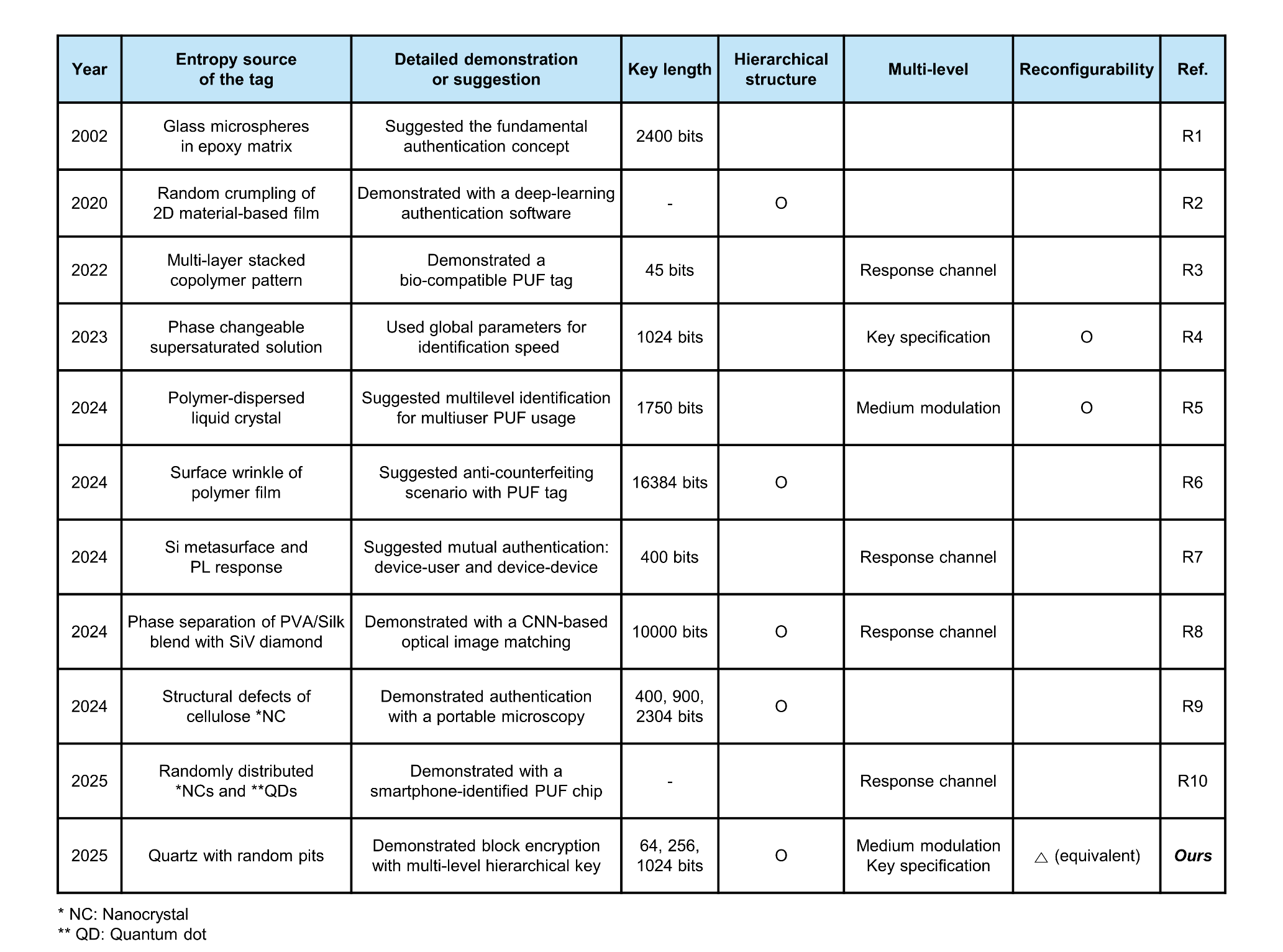
**

**Table S1. Previously reported works for PUF which related to reconfigurability, hierarchical structure, and multi-level.**

**
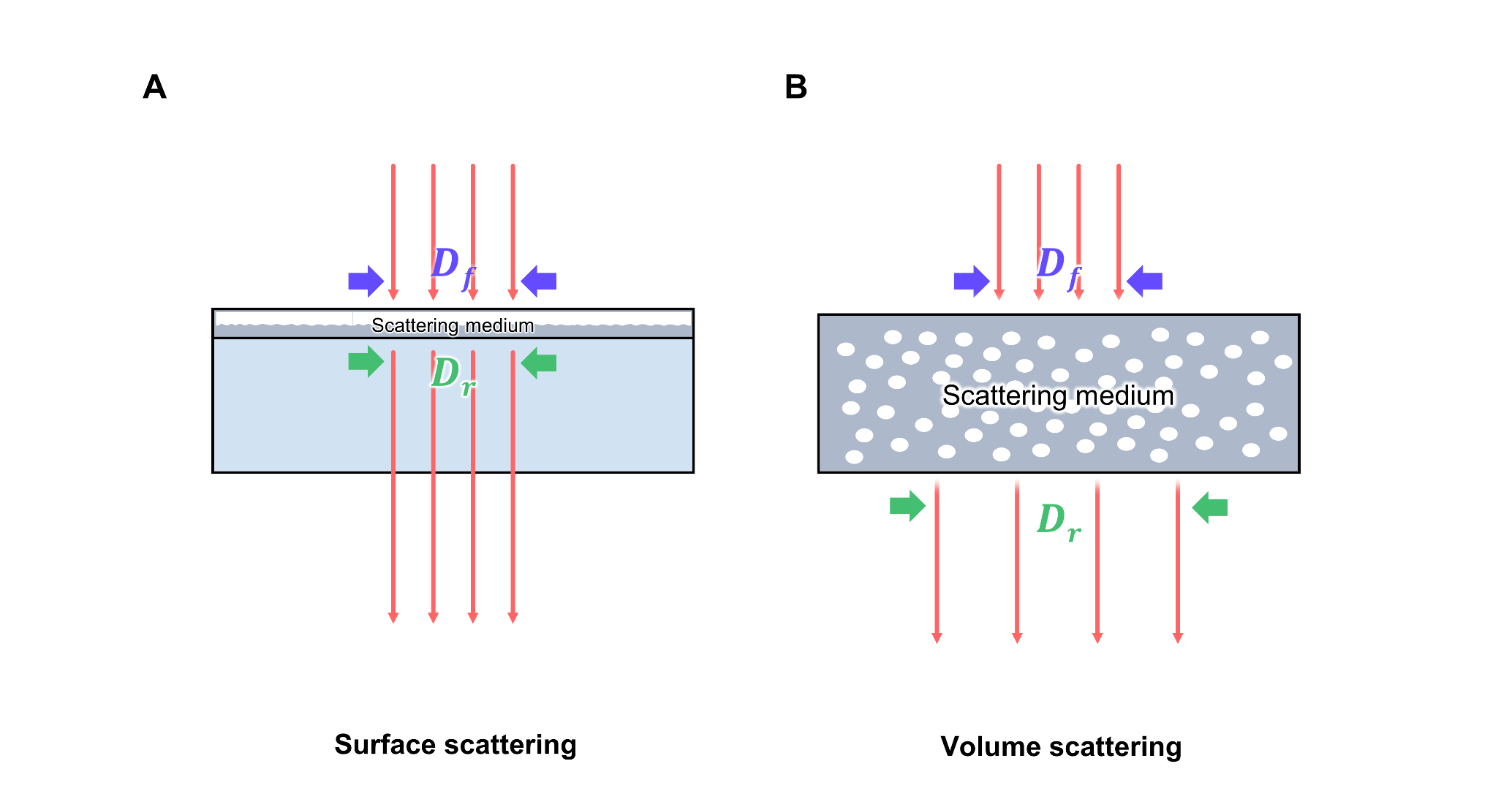
**

**Figure S1. Difference of illumination diameter in surface and volume scattering.** When determining illumination diameter to calculate the expected speckle size, the volume colored with gray is considered. Disordered structures in this domain can randomly shift the phase of light. (**A**) In surface scattering, the depth of the medium that triggers the path difference is very shallow compared to volume scattering. Therefore, the rear diameter at surface scattering is assumed to be almost equivalent to the front diameter. (**B**) In volume scattering, the illumination diameter expands due to multiple scattering within the thick medium.


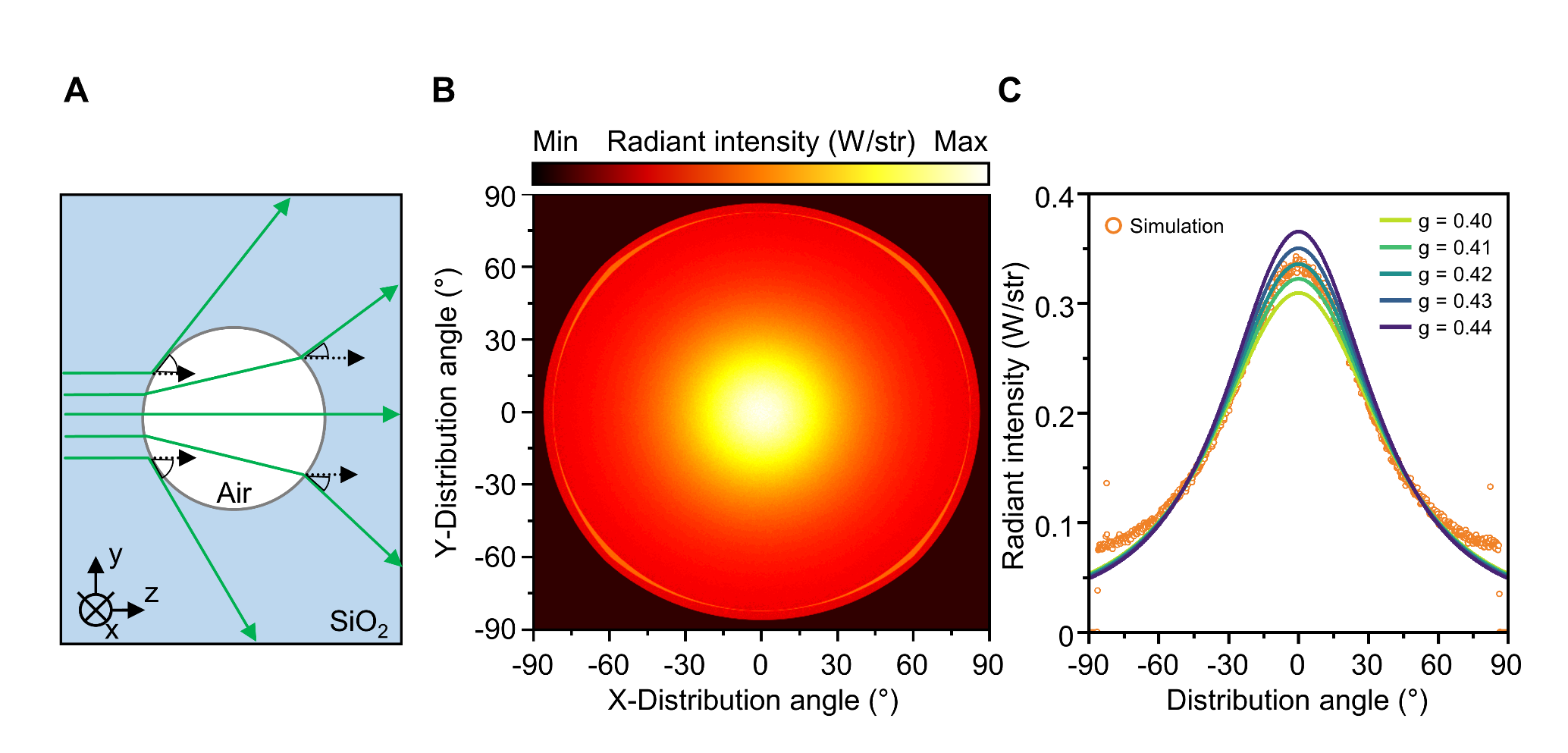


**Figure S2. Volume scattering with ray-tracing simulation.** To simulate volume scattering, we adopt Henyey-greenstein phase function that is defined as follows

$$p\left( \theta\right)=\frac{1}{4\pi}\cdot\frac{1-g^{2}}{{(1+g^{2}-2g\cos\theta)}^{3/2}}$$

where *g* is asymmetry parameter to approximate the phase function as purposed, and *θ* is angle between incident light and scattered light. (**A**) Simplified model of air sphere in the silicon dioxide substrate (*i.e.*, assumed volume scattering model). (**B**) The obtained angular distribution function for the simplified model. (**C**) Cross-sectional angular distribution at Y-distribution angle = 0 and Henyey-greenstein phase function with varying *g* parameter. According to the result, *g* = 0.42 is selected as the asymmetry parameter in conducted simulations.

**
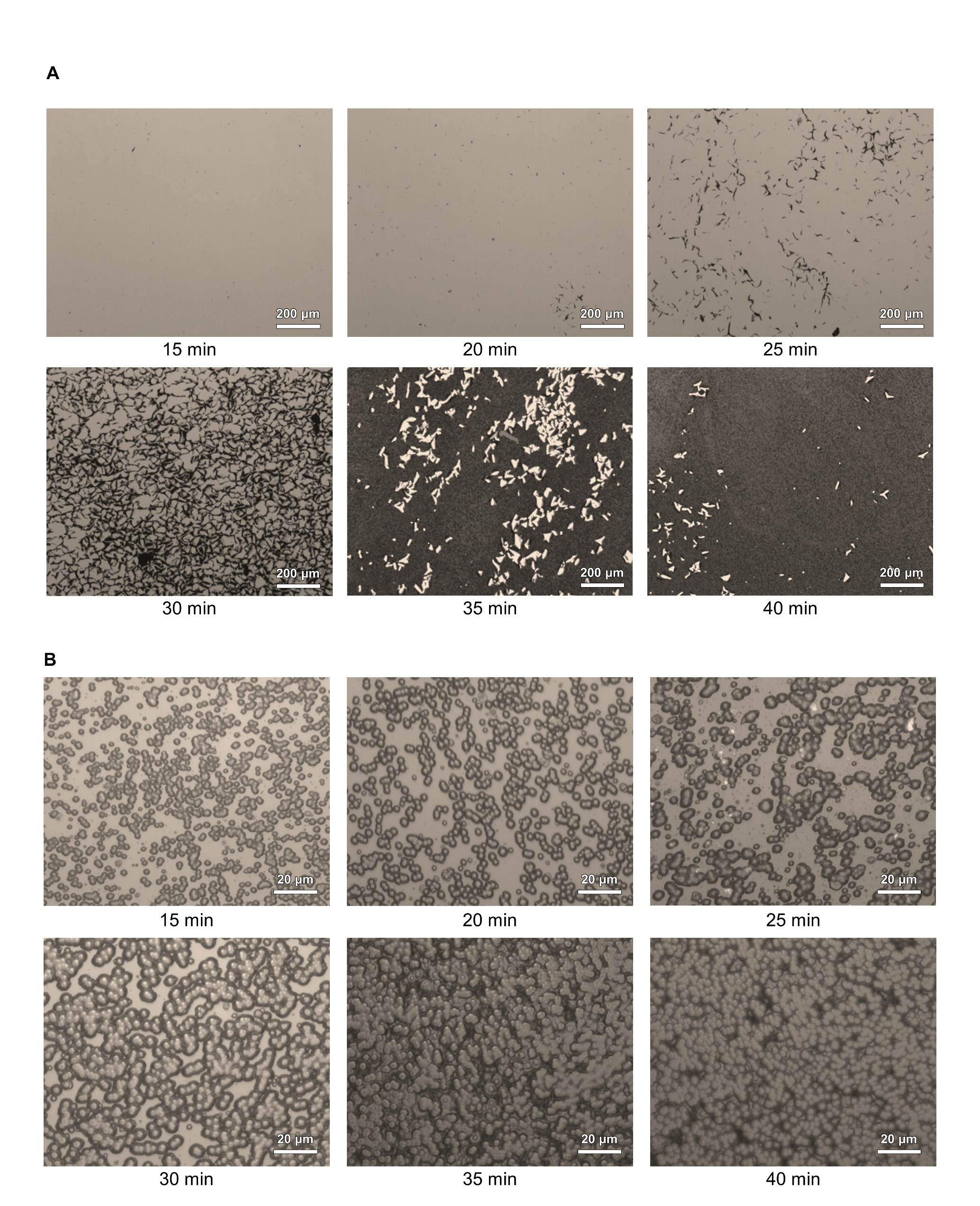
Figure S3. Optical microscopic images of RP-PUF for etching time. (A)** Before the Cr mask removal. (**B**) After the Cr mask removal. Longer etching time leads to more pit generation. At excessive etching time, there is no longer surface capacity for pit generation, and flat areas are formed due to the merger.


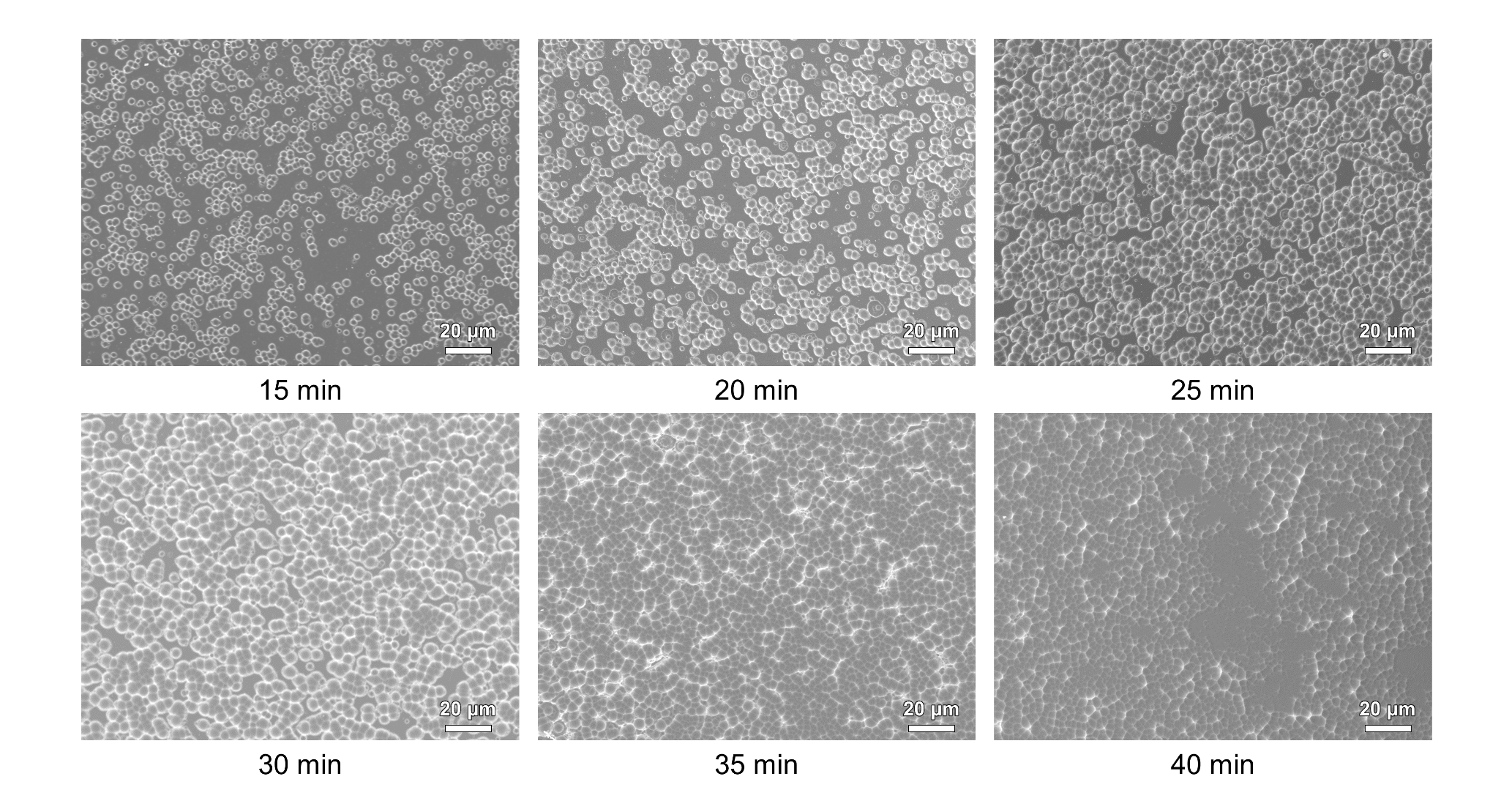


**Figure S4. Scanning electron microscopic images of RP-PUF for etching time.** The overall trend is the same as the optical microscopic images. Mergers at long etching times appear more prominent.


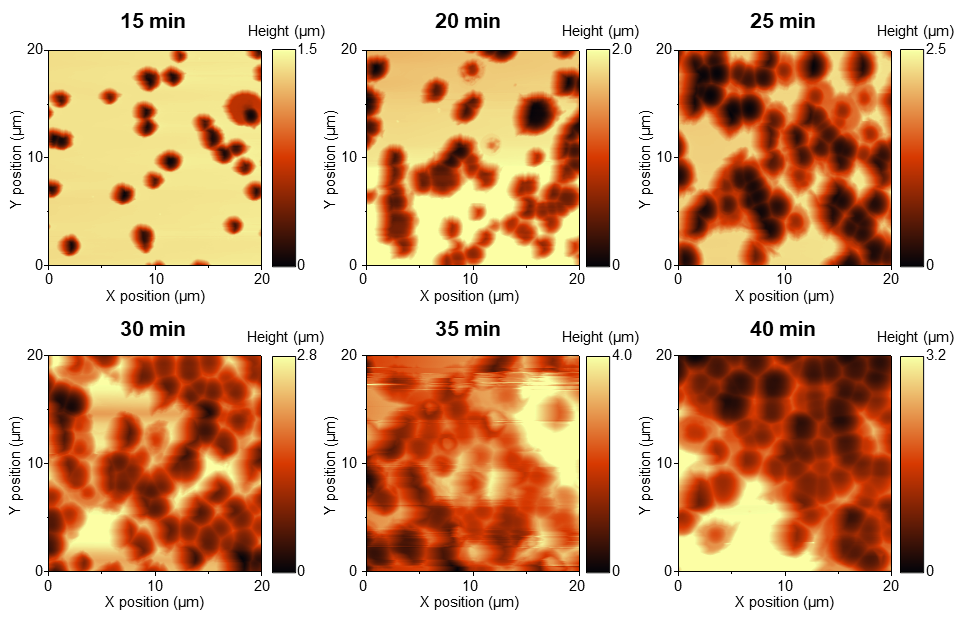


**Figure S5. Surface profile of the RP-PUF.** Atomic force microscopy (AFM) images for various etching times of 15 min to 40 min. In the case of 15 minutes, the pit profiles are collected in two separate positions due to a small amount in a single image.


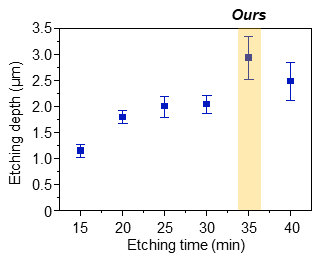


**Figure S6. Etching depth distribution for all etching times of the RP-PUF.** Etching time of 35 min shows the highest etching depth, which means the surface scattering occurs intensively. To ensure sufficient statistics, 33 pits for each etching time were selected to derive the etching depth distribution.

**
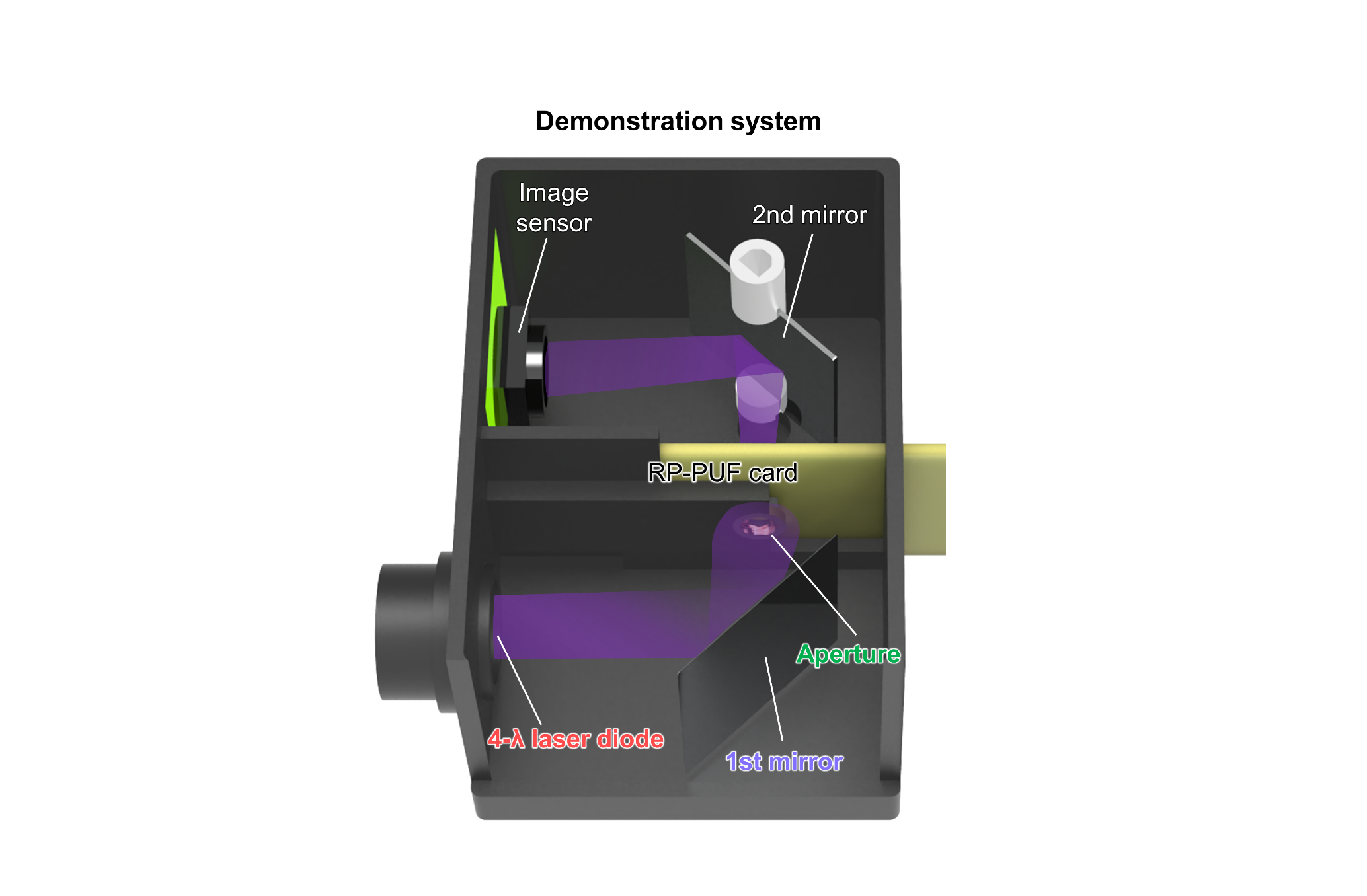
**

**Figure S7. Configuration of customized authentication system.** The demonstration system utilizes three adjustable parameters: wavelength, incident angle, and illumination diameter. Each parameter is modulated by the 4-λ laser diode, the rotatable mirror, and the aperture, respectively.

**
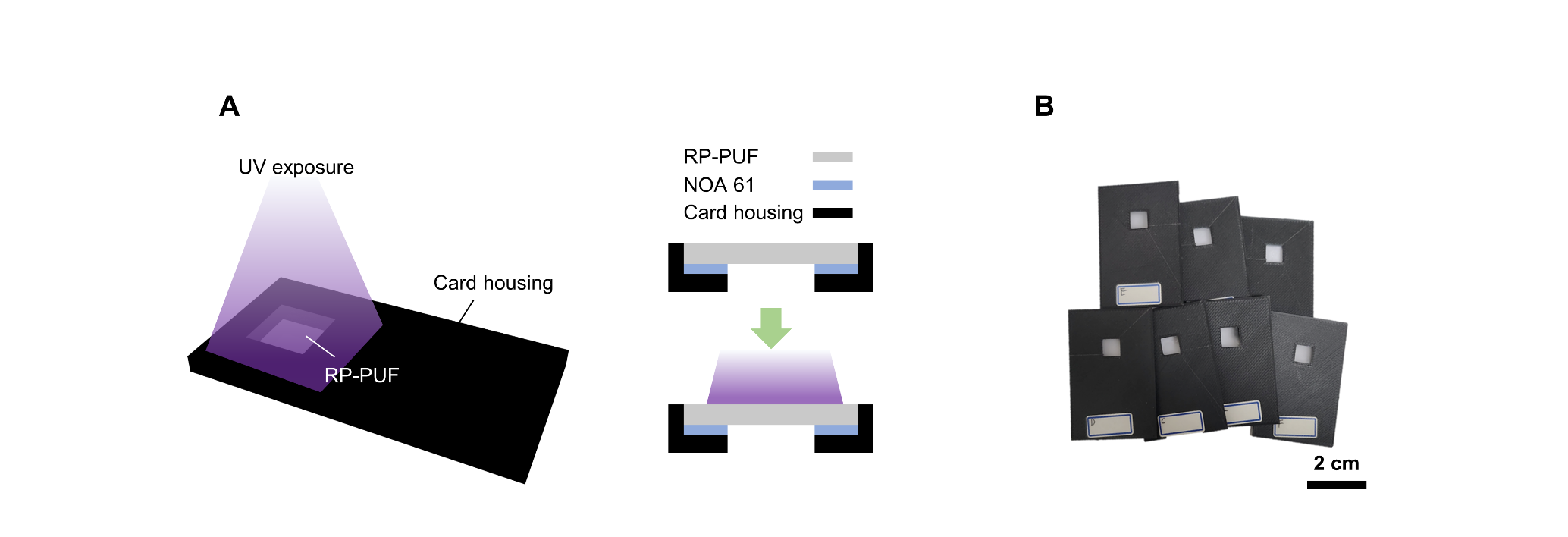
**

**Figure S8. Fabrication of RP-PUF security card.** (**A**) A 3D printer is used to produce a security card housing. The RP-PUF tag is attached using optical adhesive and cured under UV exposure. (**B**) Photograph of fabricated security cards for demonstration with authentication setup.

**
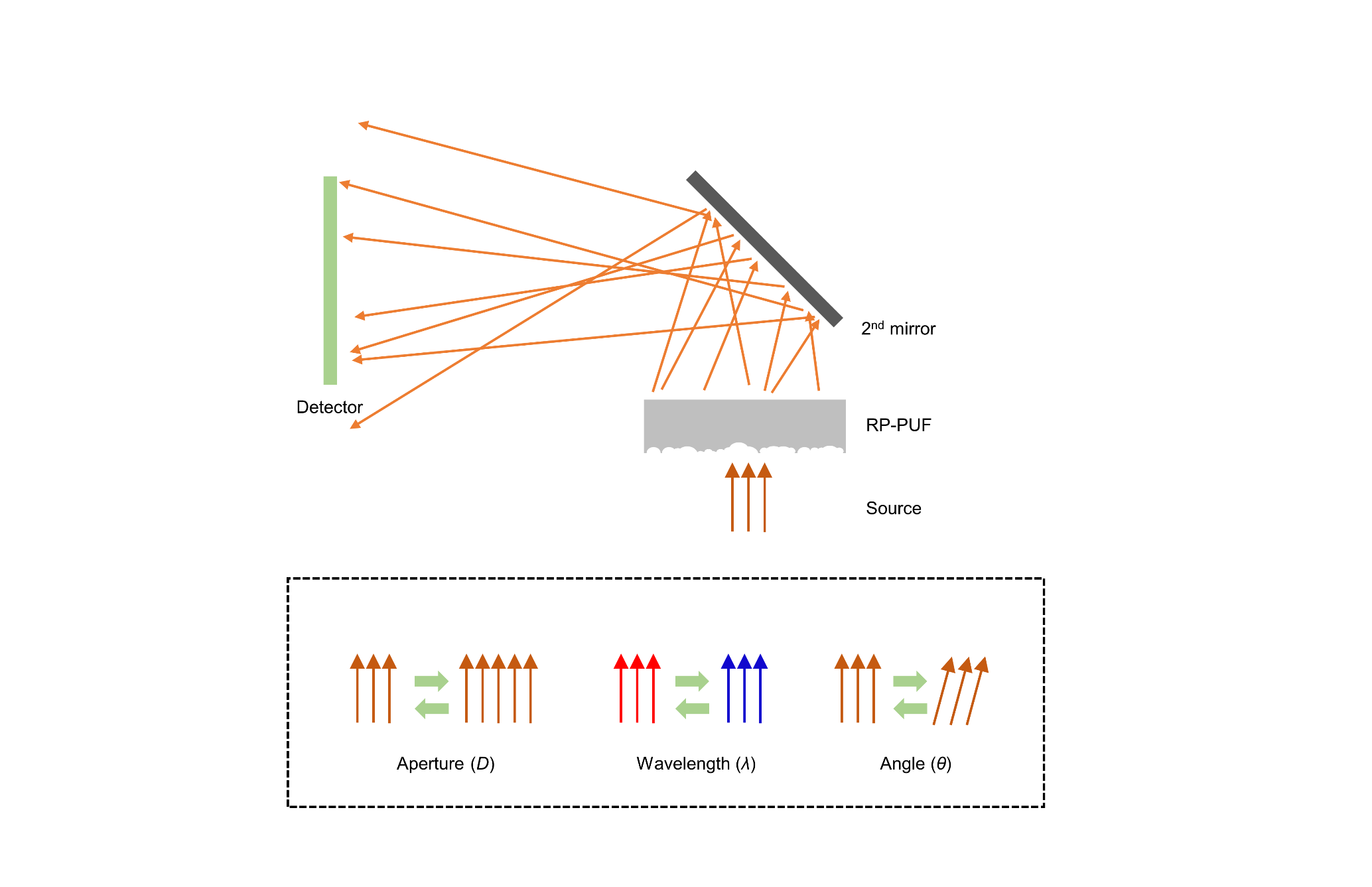
**

**Figure S9. Ray-tracing simulation schematic for validation of parameters.** Layout of the ray-tracing simulation. The system consists of a circular ray source (*i.e.*, light after passing the aperture), an imitated RP-PUF, a mirror, and a ray detector. The diameter change of the source is equivalent to the aperture diameter change, and the wavelength is also configurable. The direction change of the source emulates the first mirror rotation as the incident angle.


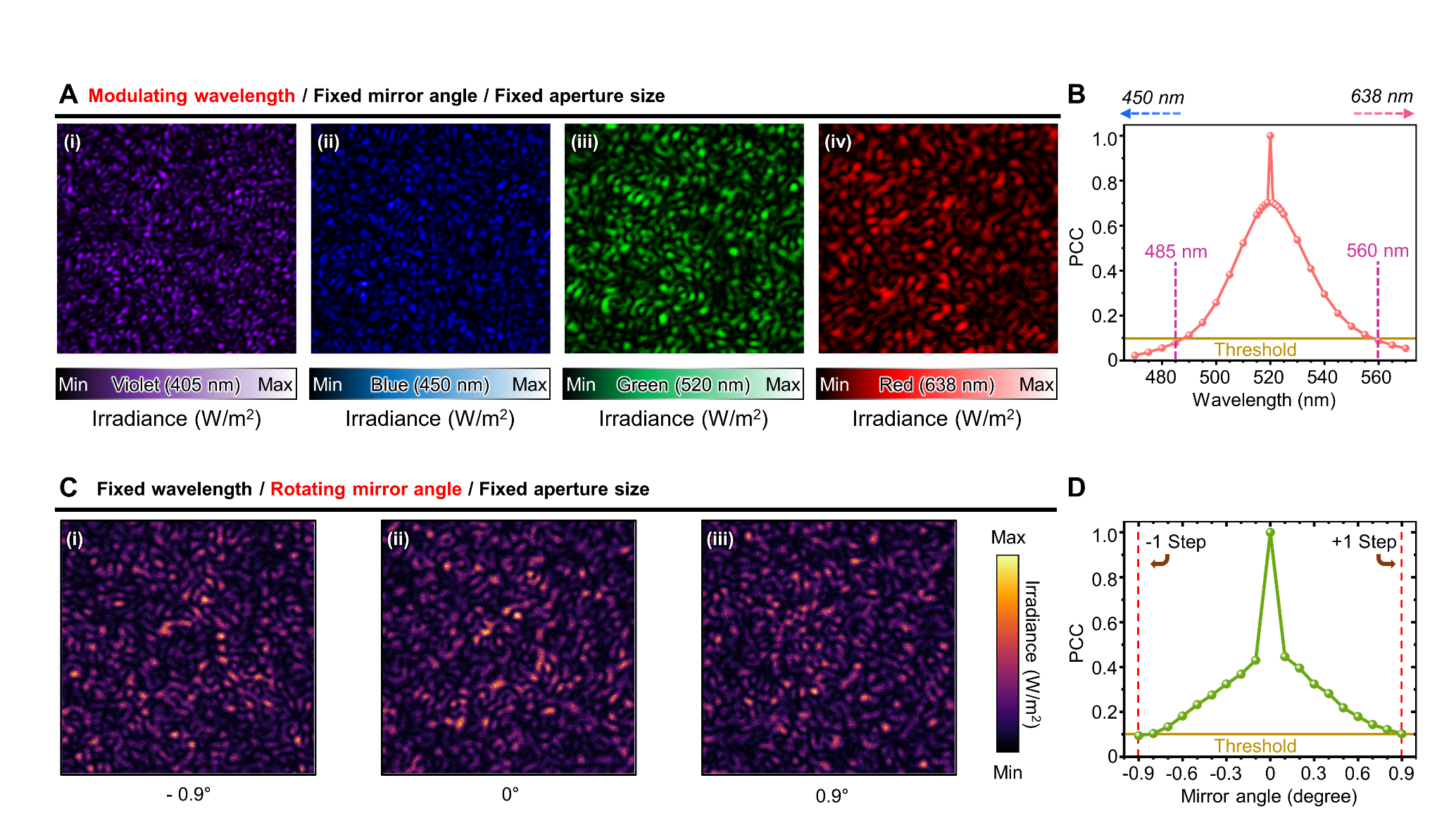


**Figure S10. Validation of key space expansion with ray-tracing simulation regarding wavelength and incident angle.** (**A**) Ray-tracing simulation results with wavelength variations. (**B**) PCC based on wavelength variation with 520 nm as a reference. The threshold for determining unique seeds is set to 0.1. The PCC shows a value lower than 0.1 at wavelengths of 485 nm or larger than 560 nm. Blue (450 nm) and red (638 nm) laser diodes are in this range. Therefore, utilizing laser diodes in this wavelength region can be expected to result in extremely low correlation of each raw image. (**C**) Ray-tracing simulation results with the incident angle changes. (**D**) PCC based on the angle changes with normal incidence as a reference. The step represents angular increments used in our experiment.


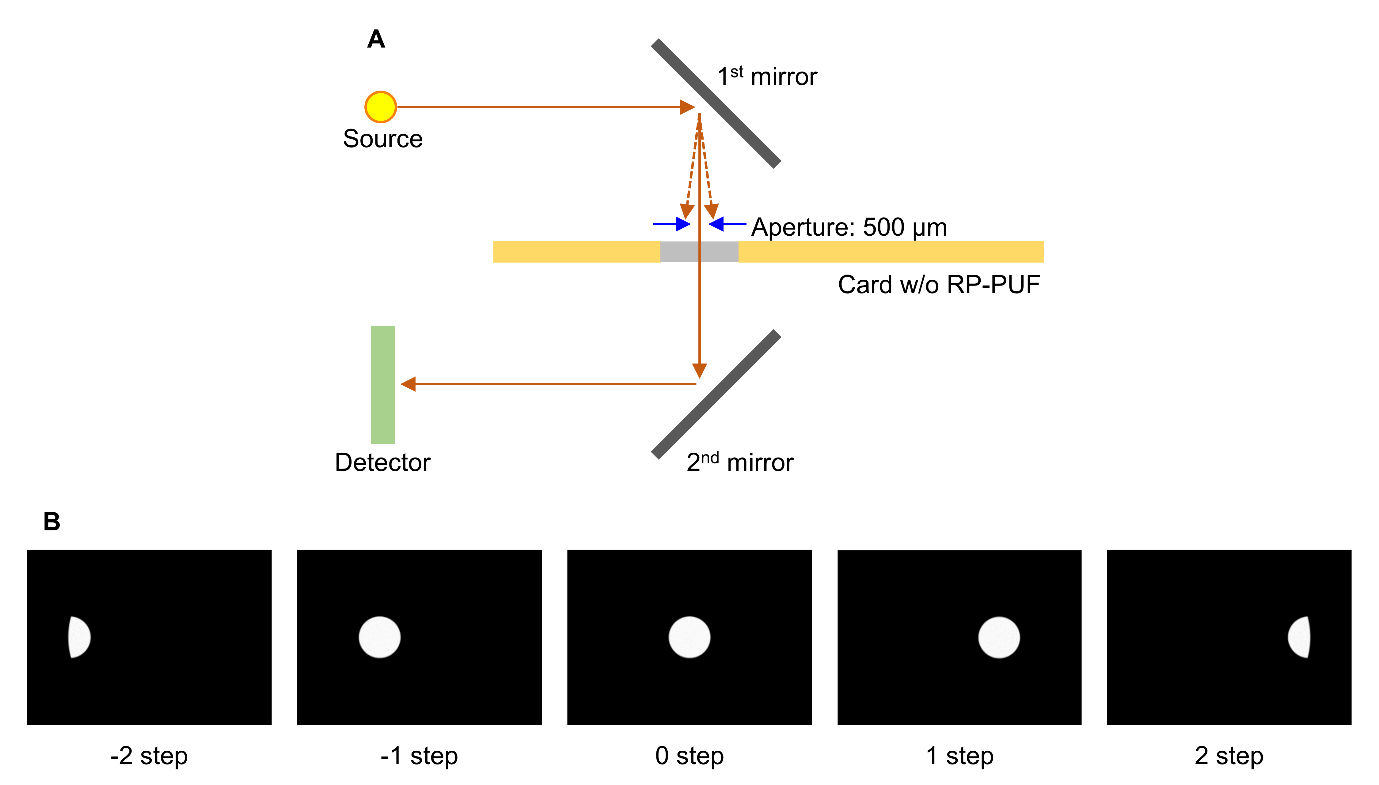


**Figure S11. Angular range evaluation directing the ballistic central beam to the image sensor.** (**A**) A ray-tracing simulation schematic for the description of used incident angle range used in this work. The aperture size is set to 500 μm. As the first mirror rotates, the central beam is partially blocked following the path indicated by the brown dash. (**B**) Detector images for the proposed steps. A single step is 0.9 ° rotation of the first mirror. If the mirror is rotated more than two steps, the central beam will be completely blocked. Therefore, we use the angular range from -2 to 2 in steps.

**
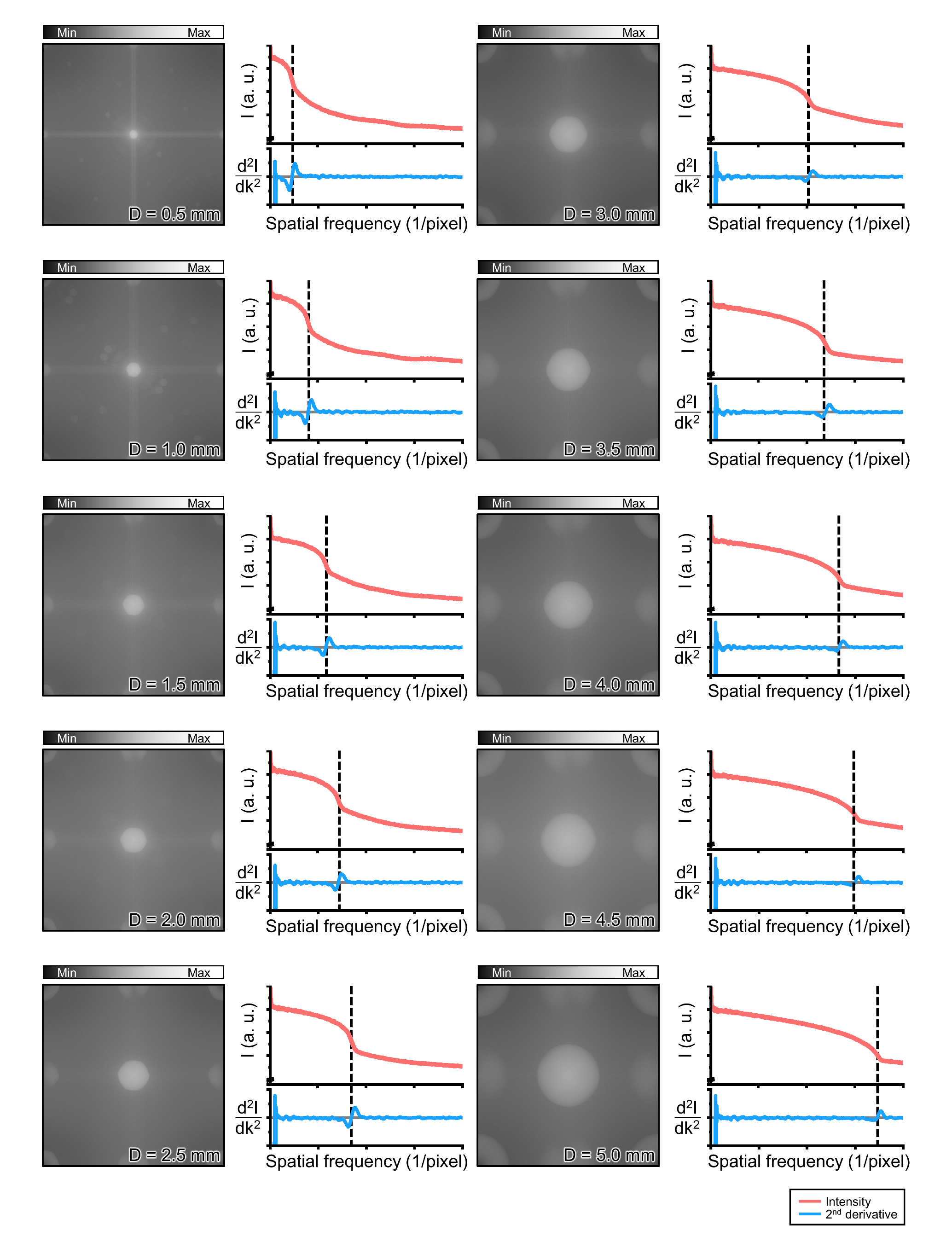
Figure S12. Spatial frequency distributions by 2D-Fourier transform.** As the aperture grows, the intensity distribution radiates from the center, presenting a high spatial frequency contribution in the speckle.

**
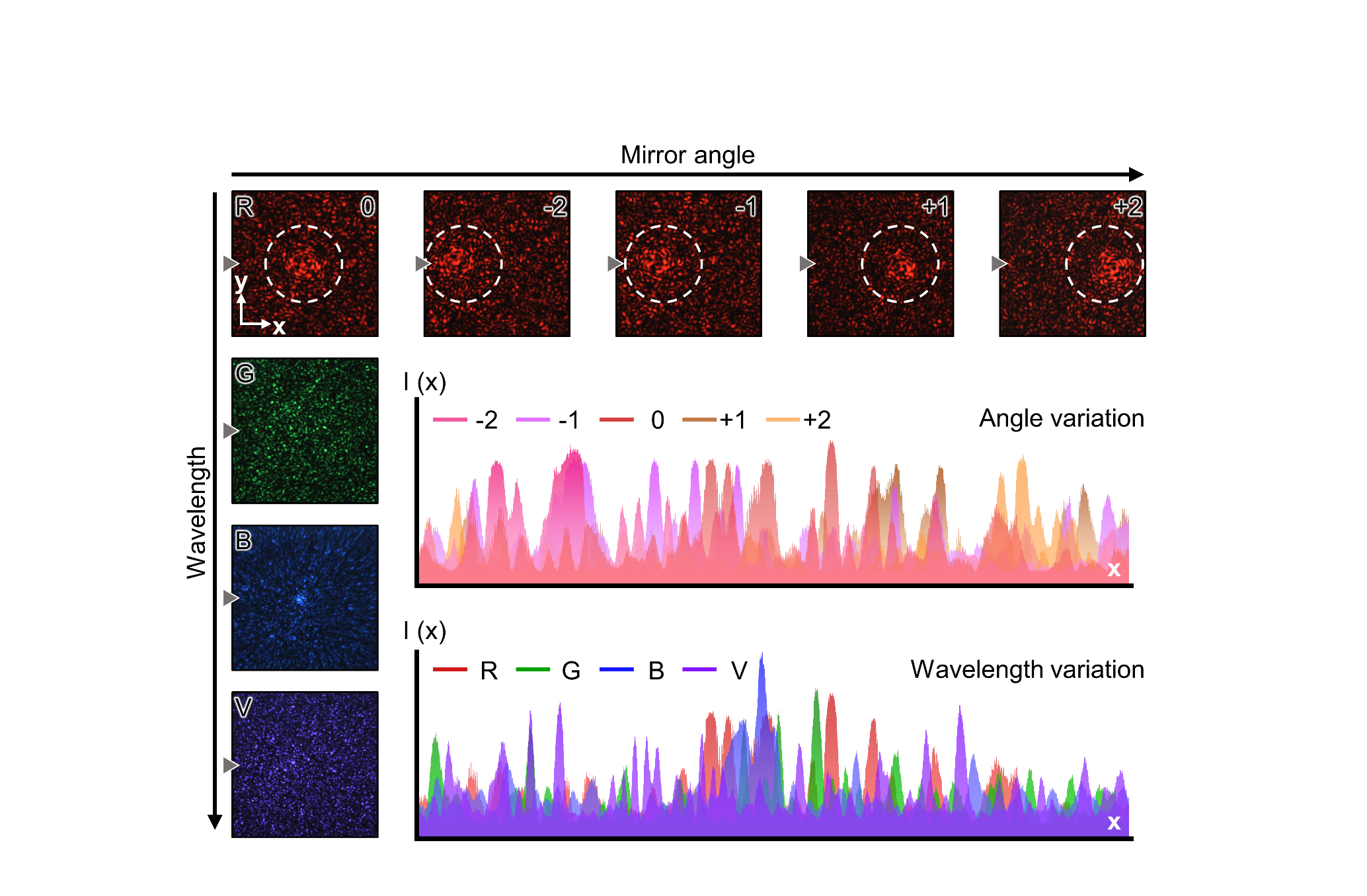
Figure S13. Experiment of key space expansion based on wavelength and incident angle manipulation.** Unique images acquired with varying wavelengths and mirror angles. The white dashed circle shows the change in light direction. The filled line plots are drawn with the central row of images marked with the gray triangle.

**
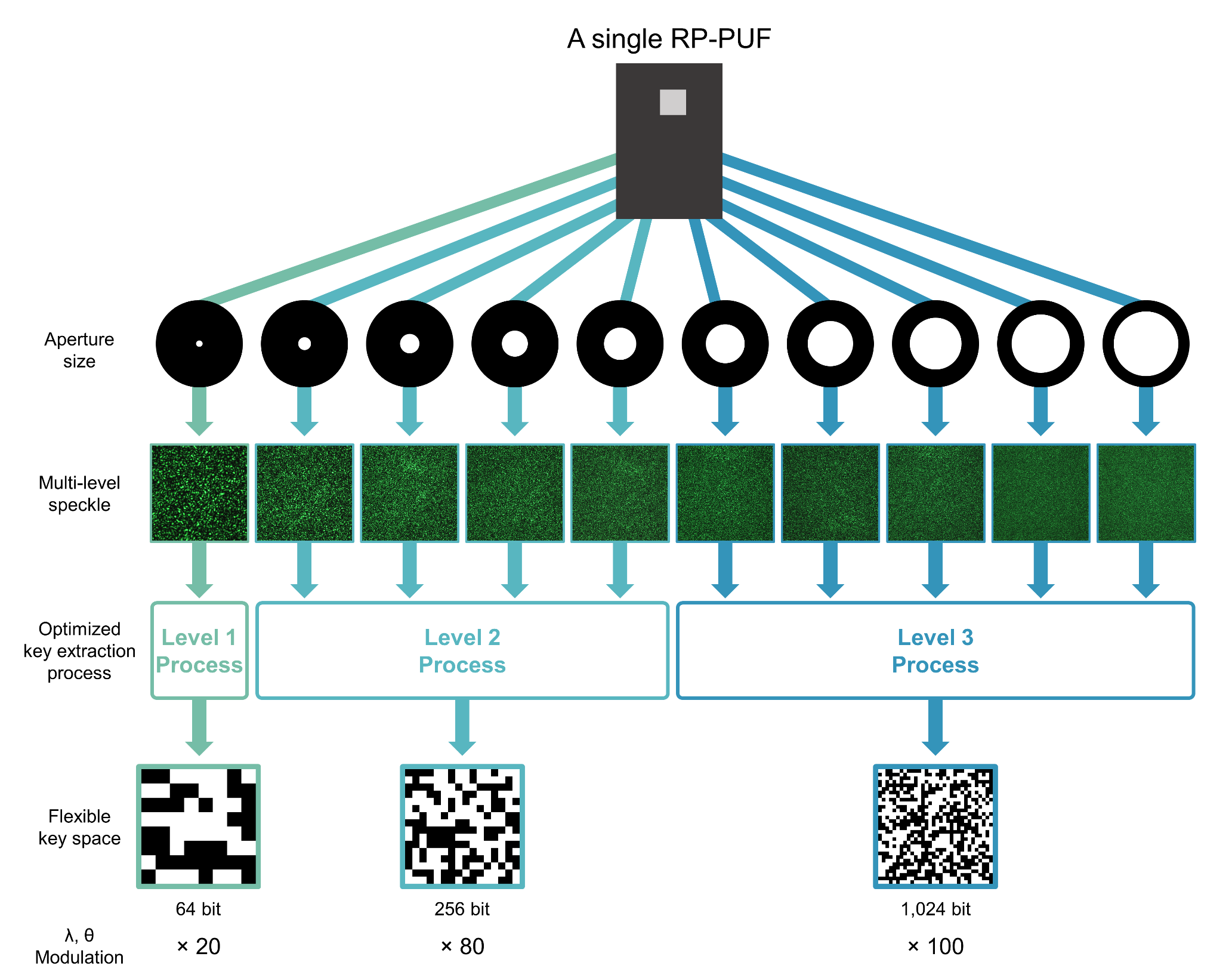
**

**Figure S14. A schematic of aperture size adjustment for multi-level speckle generation and various key specifications.** Adjusting the aperture size allows RP-PUF to provide speckles of different sizes. By applying an optimized key extraction process for speckle size, various-length keys are obtained. Each level is assigned one, four, and five aperture steps in increasing order. These aperture steps are multiplied by four wavelengths and five incident angles, resulting in 20, 80, and 100 keys for each level, respectively.


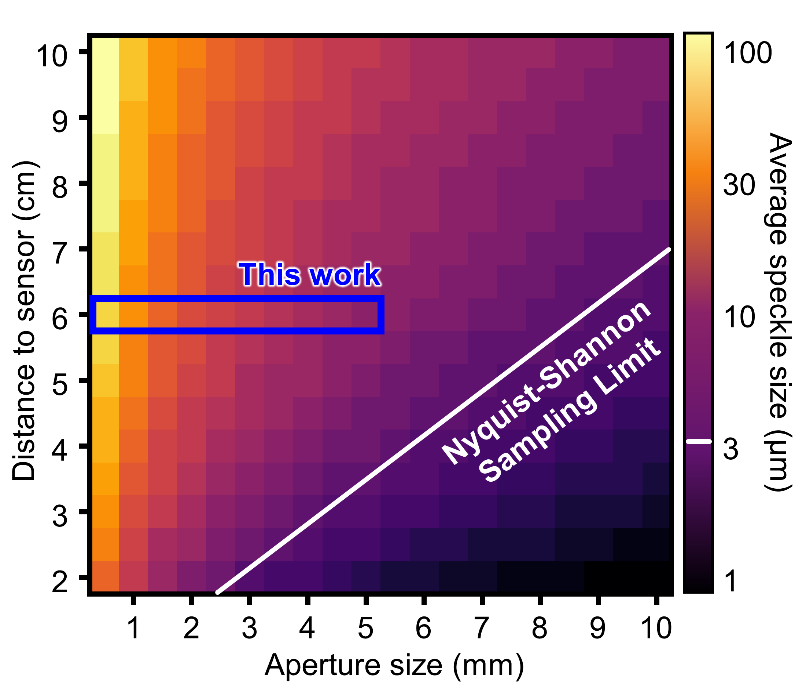


**Figure S15. Nyquist-Shannon sampling limit for system configurations.** As the aperture size increases and the distance to the sensor decreases, the average speckle size falls below the minimum of the Nyquist-Shannon sampling size determined by twice the image sensor pixel size. The region under the limit is unusable due to aliasing and unsuitable for PUF key generation. The white line indicates the limit, and the blue box indicates the proposed range in this work.

**
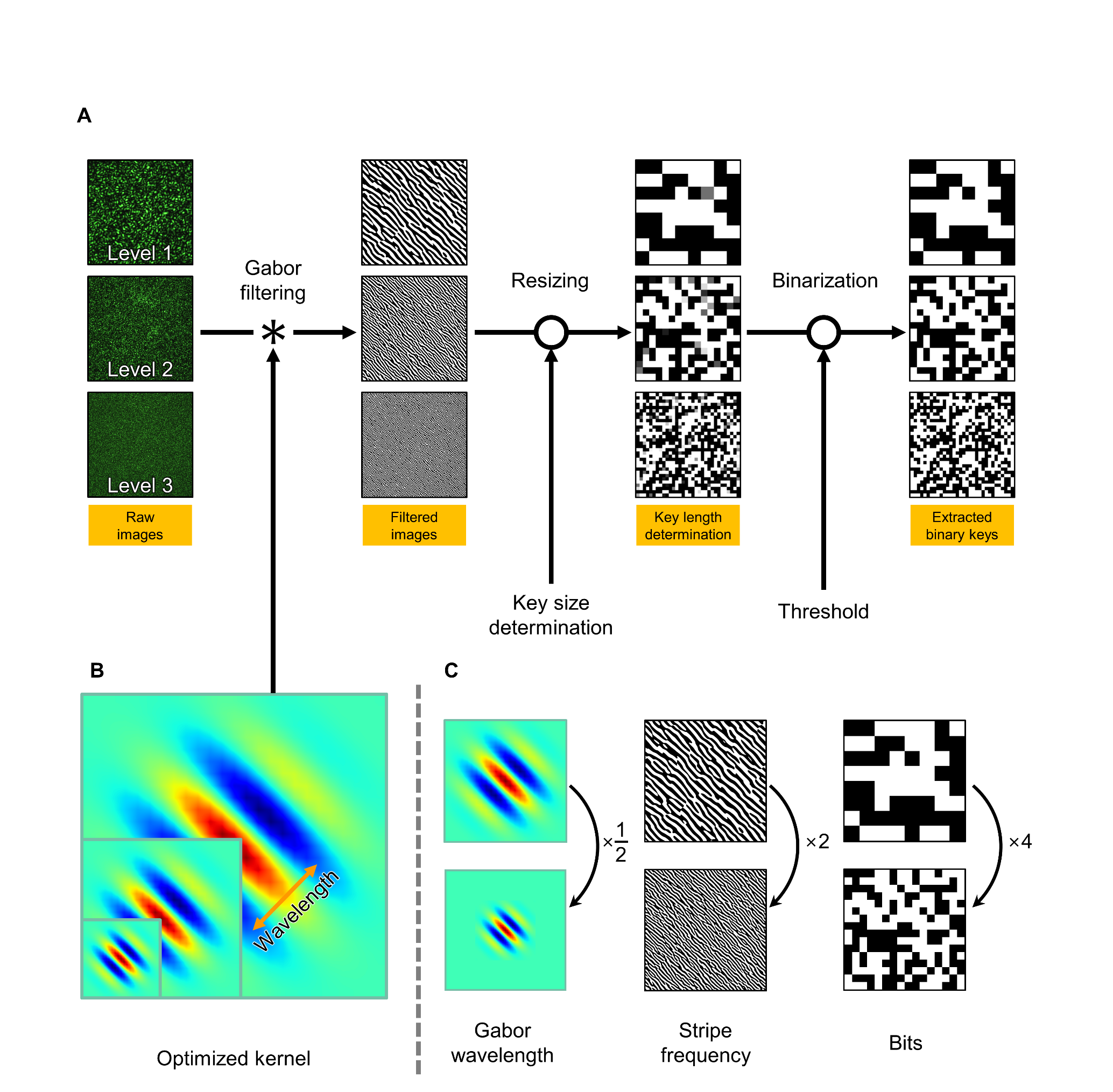
Figure S16. Gabor filtering for hierarchical key extraction.** (**A**) Key extraction process. At first, raw speckle images are filtered into random stripes by applying the Gabor kernel. Subsequently, filtered images are resized according to each level to generate sufficiently long keys. This step determines key sizes as 64-bit for level 1, 256-bit for level 2, and 1,024-bit for level 3. Lastly, an adaptive thresholding binarizes keys at all levels to be used in communication protocols, while maintaining high entropy. (**B**) Gabor kernels with various Gabor wavelengths specialized to match speckle in each level. (**C**) Gabor wavelength affects the extractable entropy of the processed stripe. At the higher level, halving the Gabor wavelength doubles the 1D stripe frequency, quadrupling the extractable bits in 2D space due to the squared relationship. This scalability of bits directly supports the proposed multi-level key system.


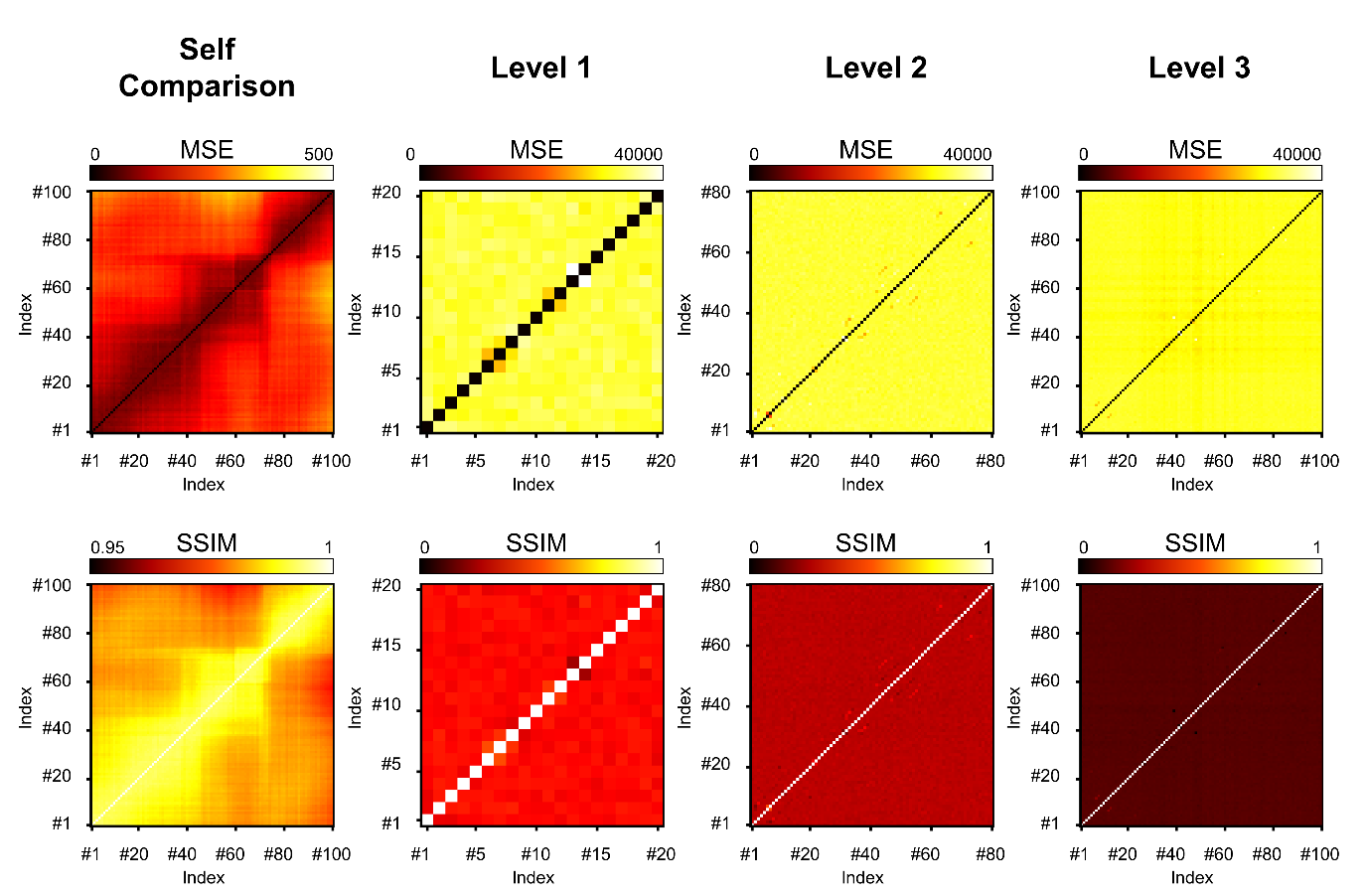


**Figure S17. Mean Square Error (MSE) and Structural Similarity Index (SSIM) of RP-PUF response after Gabor filtering.** For MSE, a value closer to 0 indicates greater similarity between images. In contrast, SSIM ranges from 0 to 1, where a value closer to 1 represents highly similar images, while a value closer to 0 indicates completely different images. Self-comparison, indicating similarity between 100 Gabor-filtered images obtained using the same aperture size, wavelength, and incident angle, exhibits high similarity because they are identical except for noise. On the other hand, level 1, 2, and 3 results reveal that no similarities are detected across all parameters.

**
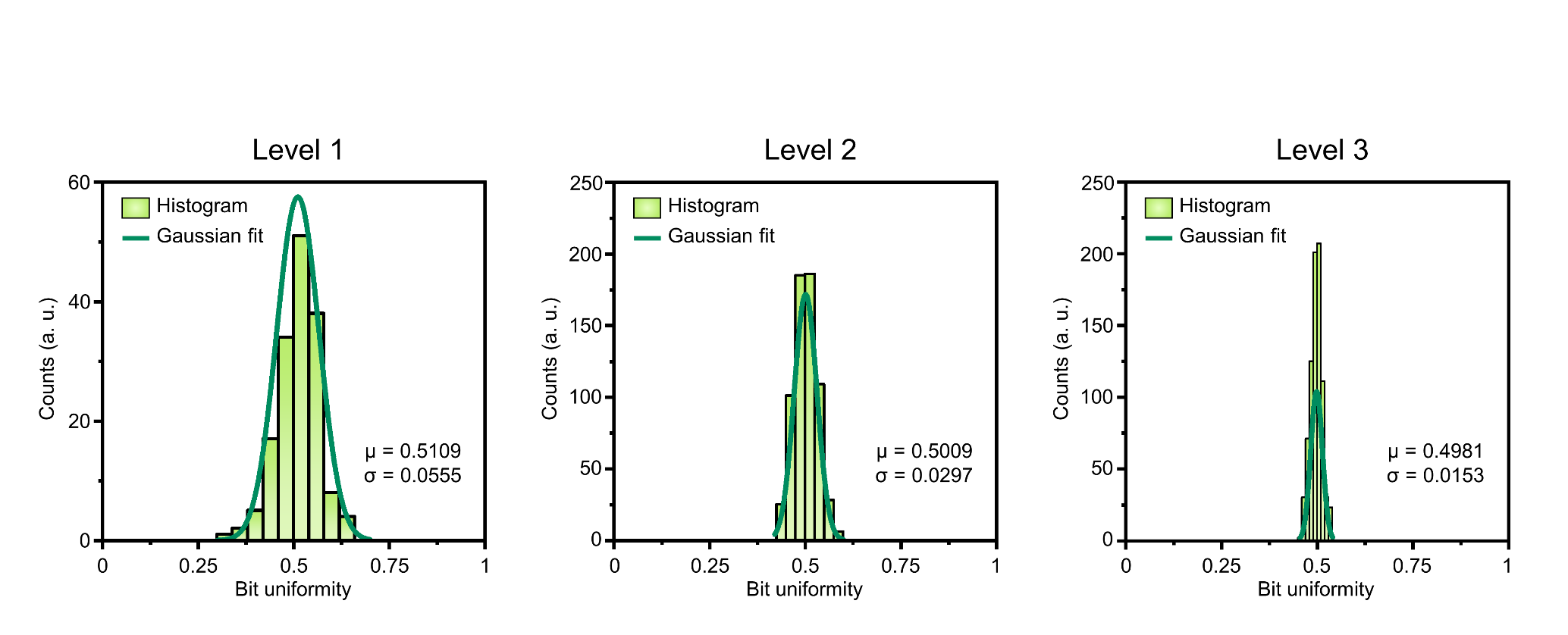
**

**Figure S18. Uniformity distributions of RP-PUF system for 3 levels.** The mean values for all levels are near 0.5, signifying the RP-PUF system produces non-biased bit arrays.

**
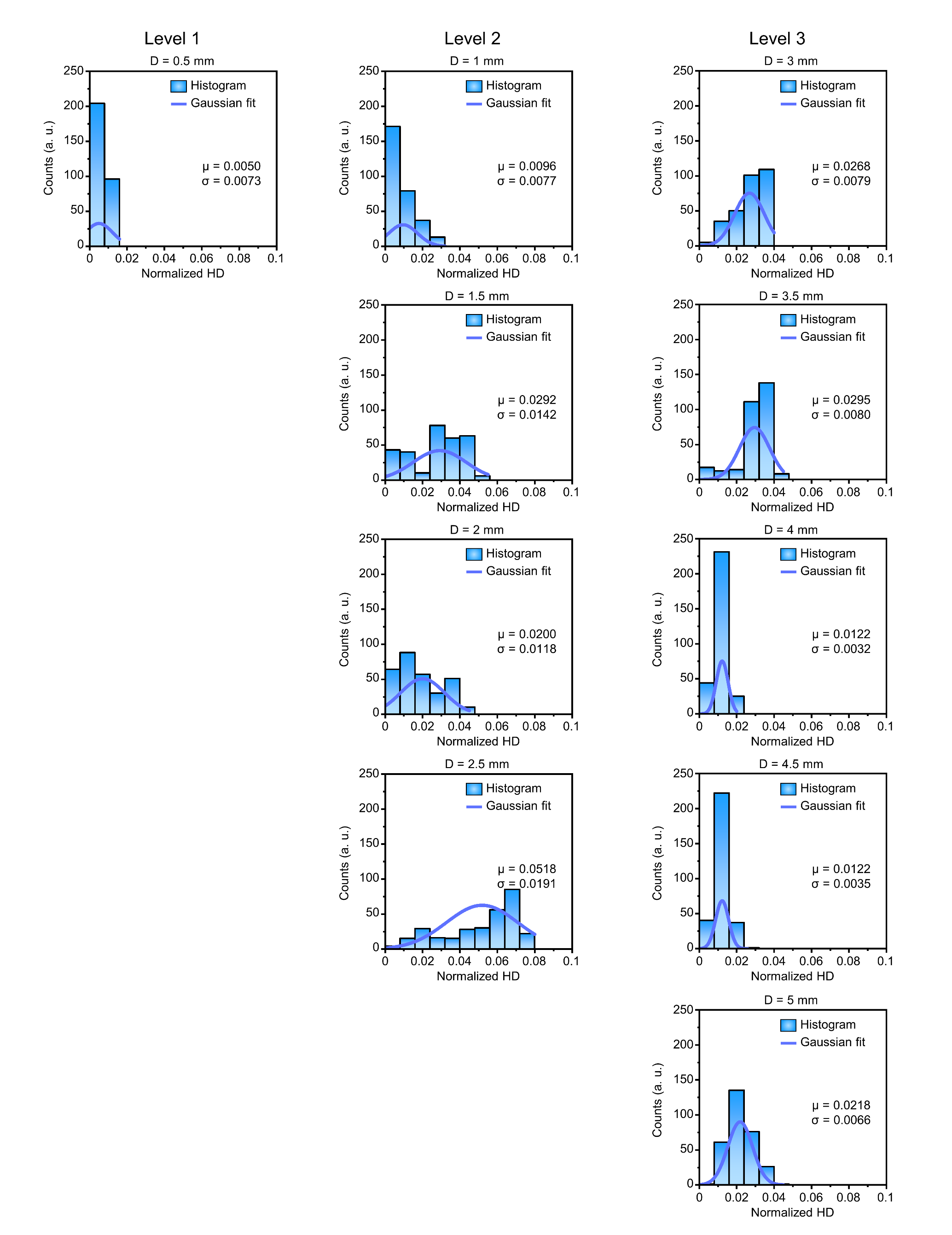
**

**Figure S19. Intra-HD distributions of RP-PUF system for 10 aperture sizes.** The mean values for all aperture sizes are almost 0 of the ideal. Intra-HD is measured separately for each aperture size because it is an iterative self-comparison under a fixed configuration.


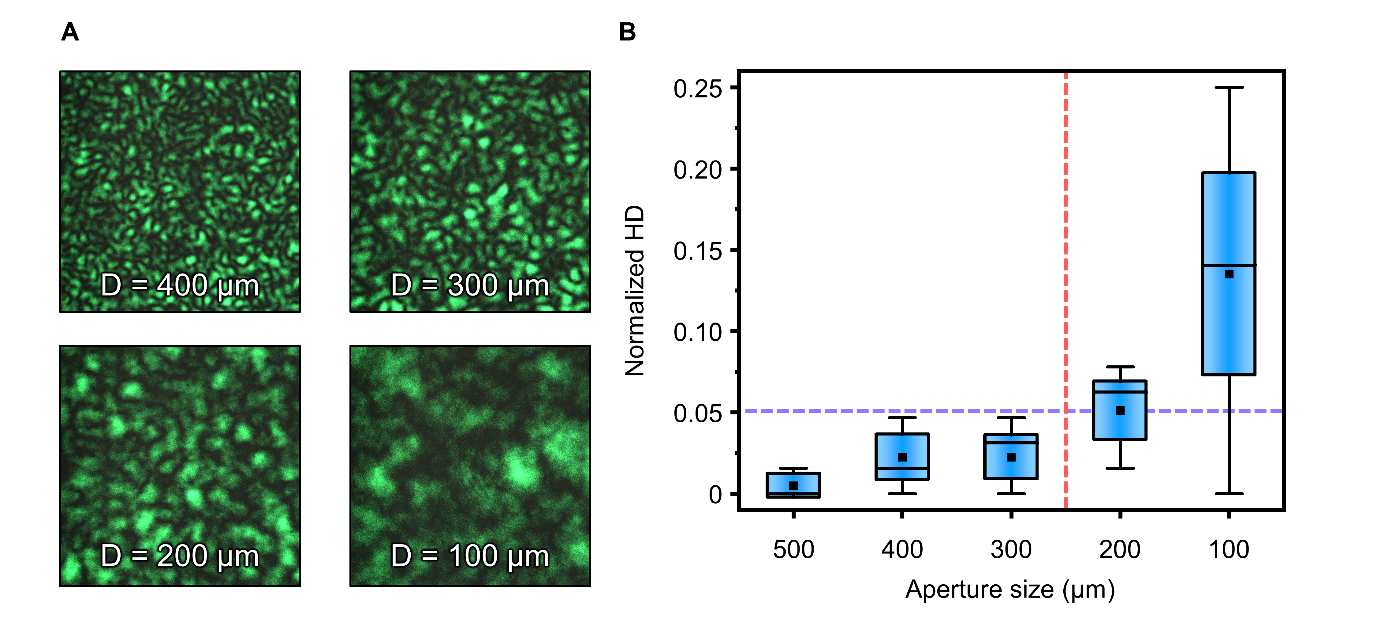


**Figure S20. Intra-HD characteristic with aperture sizes below 500 μm.** (**A**) Raw speckle images for 400, 300, 200, and 100 μm. (**B**) Intra-HD of 300 repetitive self-comparisons for each aperture size, with 500 μm as reference. Keys are extracted using the optimized “level 1” process. Increasing instability of HD exceeding 0.05 after 300 μm indicates the necessity of a newly optimized key extraction process. However, decreased structural peaks in raw images renders them unsuitable for key generation. Also, to effectively exploit the aperture region below 500 μm, a circular zero-aperture iris and noise countermeasure against increasing exposure time are prerequisites.


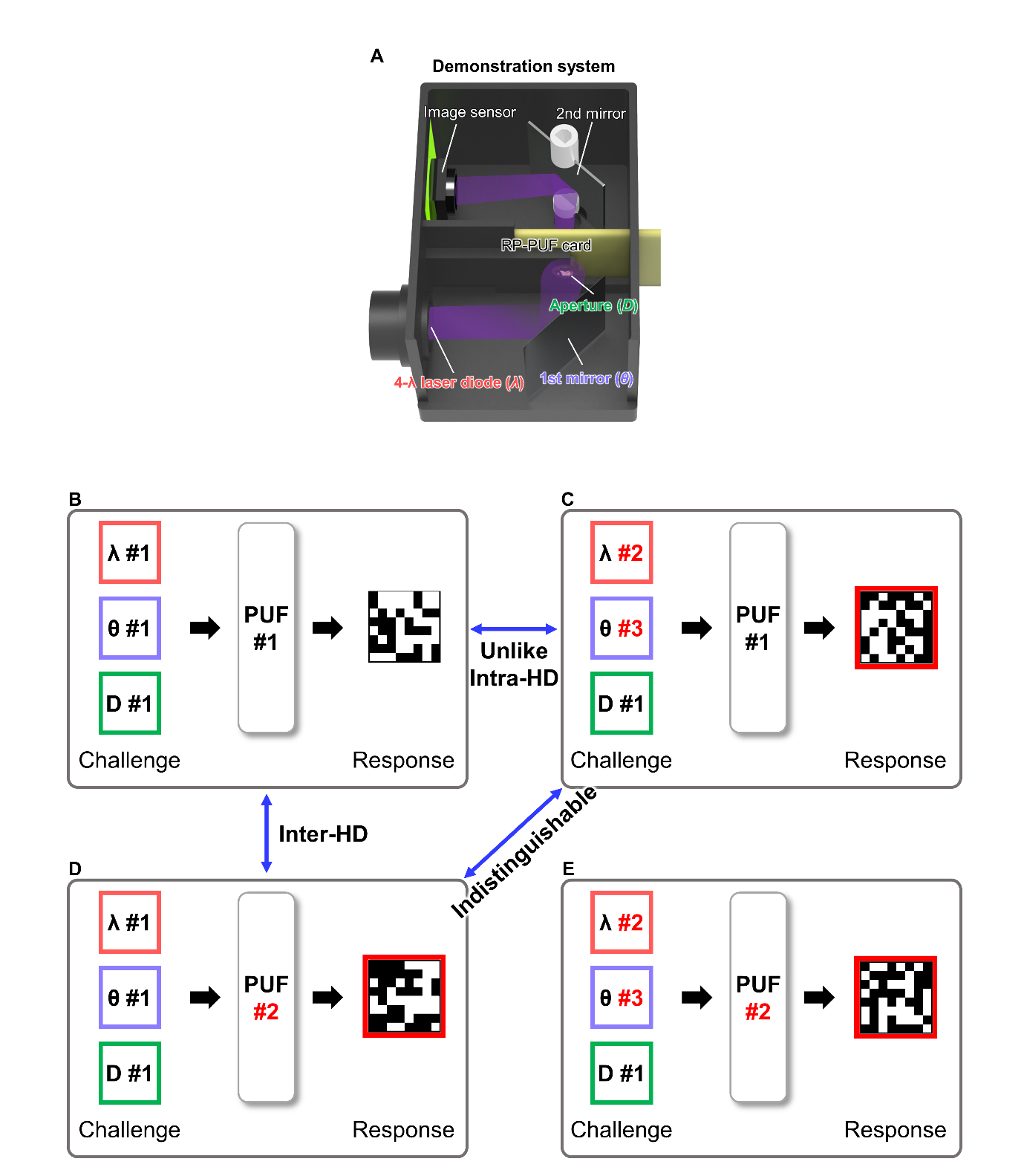


**Figure S21. Response scenarios for level 1 to construct ‘Unlike-HD’ consisting of inter-HD, unlike intra-HD, and dual conditions.** (**A**) Overview of and elements contributing to key space expansion with corresponding parameters in the proposed system. (**B**) Basic condition to generate a key at the RP-PUF system. (**C**) Challenge modulation with the same PUF instance condition for unlike intra-HD comparison. (**D**) A different PUF instance condition for inter-HD comparison. (**E**) Dual condition with the different PUF instance and challenge modulation. Within a strong PUF, the modulation of challenge parameters can be equivalent to an independent tag instance. Therefore, HDs with (**C**), (**D**), and (**E**) are grouped as ‘Unlike HD’, representing uniqueness for determining the authentication threshold.


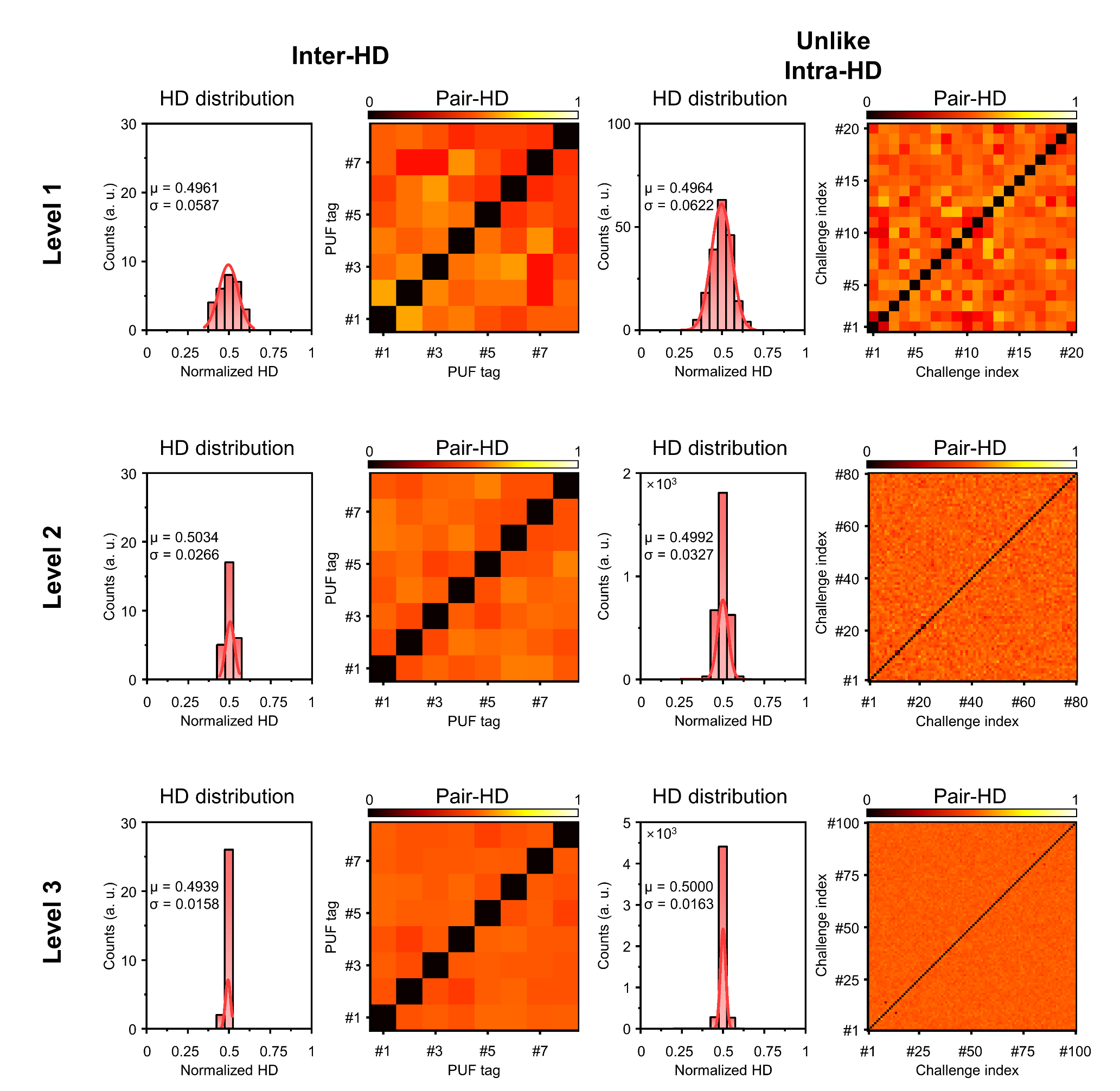


**Figure S22. Inter-HD and unlike intra-HD distribution of RP-PUF.** Given that both inter-HD and unlike intra-HD are distributed around 0.5, responses from the dual condition can be considered as an independent PUF instance.

**
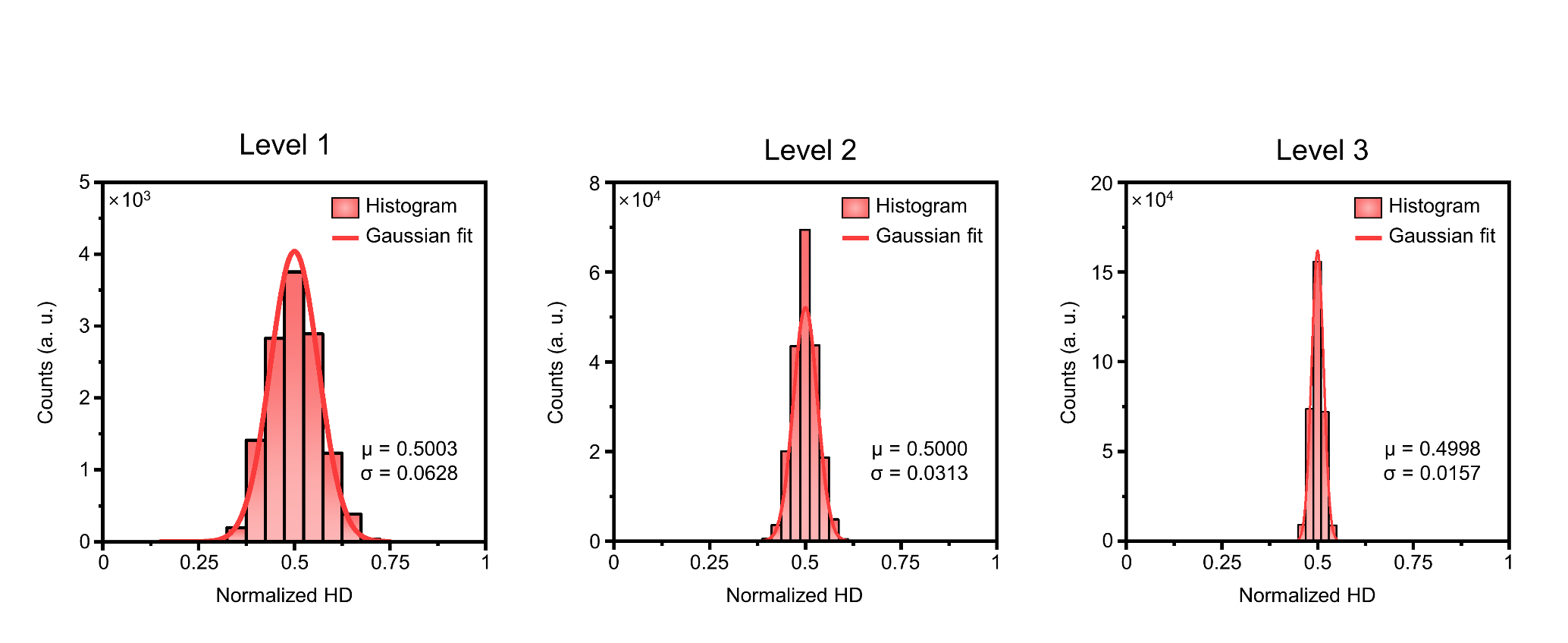
**

**Figure S23. Unlike HD distributions of RP-PUF system for 3 levels.** The unlike HD consists of unlike intra-HD, inter-HD, and dual condition. If there are no relations between bits, the HD between two binary arrays follows the binomial distribution Bin (n, p), where n is the length of the array and p is 0.5 in the binary system. In case of normalized HD divided by n, the mean of HD is n×p/n =p while the standard deviation of HD is √(n×p×(1-p))/n. In our case, binomial standard deviations are 0.0625, 0.03125, and 0.015625 for each level with corresponding n of 64, 256, and 1,024. For all levels, the mean values are near 0.5, and the standard deviation approaches that of a binomial distribution.

**
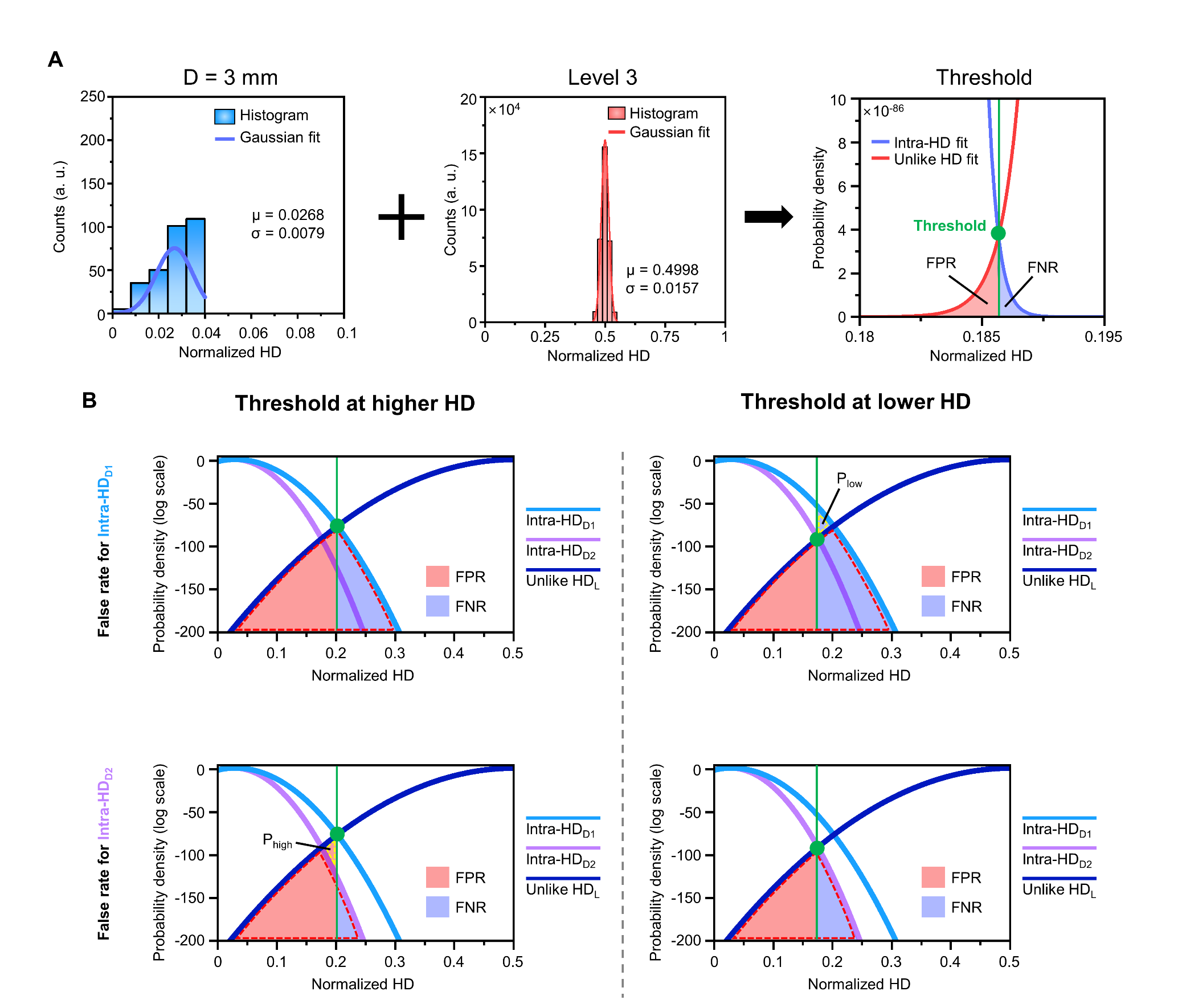
**

**Figure S24. Authentication threshold determination and consideration of multiple aperture sizes.** (**A**) Normal distribution curves are extracted based on the intra-HD of a specific aperture size and the corresponding unlike HD of the corresponding level. Then, both graphs are plotted to obtain the threshold HD derived from the intersection. This point represents the minimum probability of incorrect authentication (*i.e.*, the sum of false-positive and negative rates). (**B**) Thresholds at the level comprising several aperture sizes. Multiple thresholds can exist that minimize the probability of false authentication for each unlike HD. To determine the better threshold, we compared false rates in both cases. The areas indicated with a red dash are common in both cases and therefore do not need to be considered. Given the graph on the log scale, P­_high_ is substantially lower than P_low,_ indicating a lower probability of false authentication. Therefore, the threshold at the higher HD (*i.e.*, the rightmost value) is determined as a global threshold for the level.

**
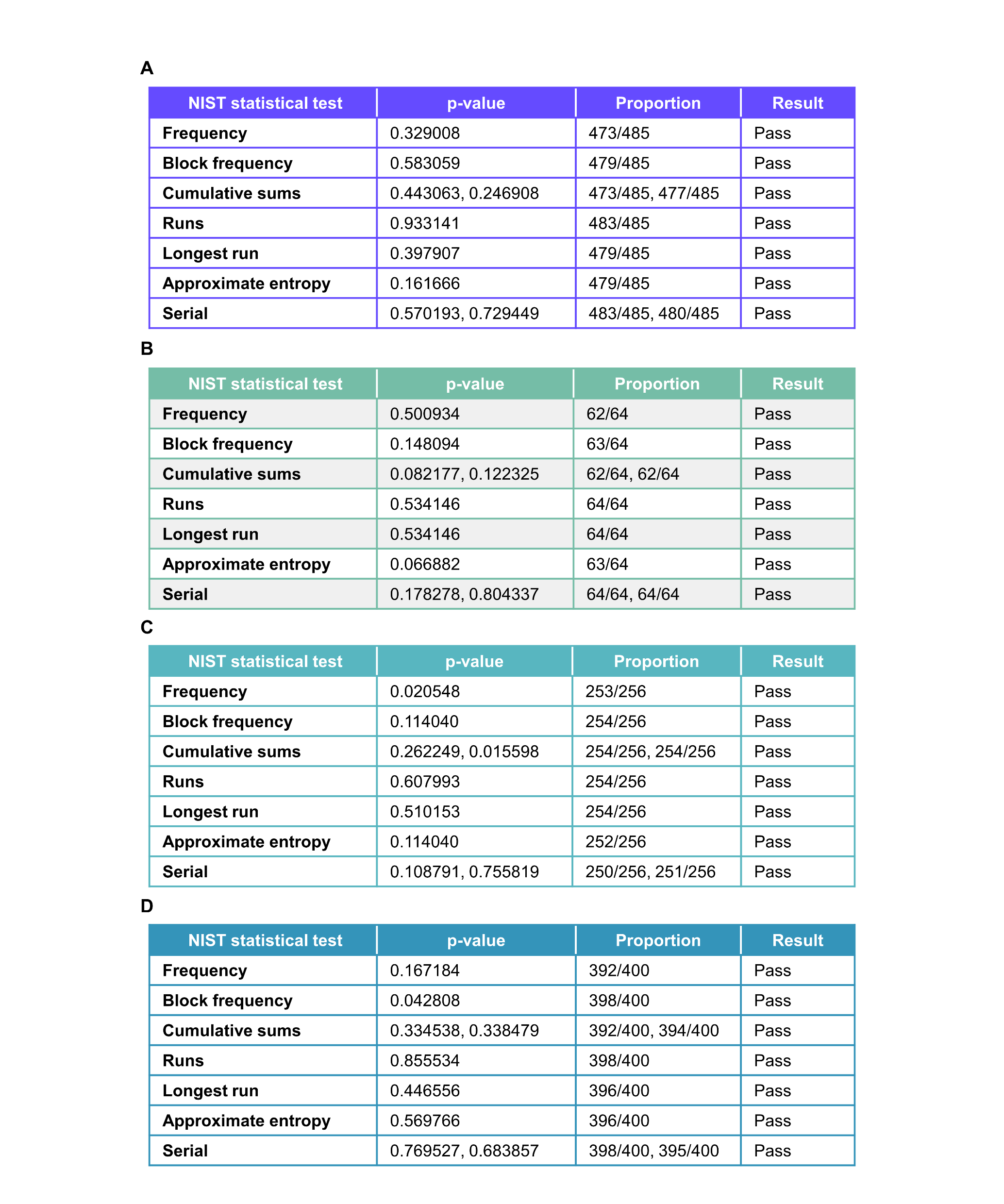
**

**Table S2. NIST 800-22 randomness test.** Bitstreams are collected from 1,600 keys from 8 different RP-PUFs. The bitstreams are considered random only if the *p*-value ≥ 0.01 and exceed the proportion threshold rate. (**A**) The collection of all levels comprising 485 sequences of 2,048 bits (total 993,280 bits) and a threshold rate of 473/485. Subsequently, the NIST SP 800-22 test is performed for bitstreams of each level separately. (**B**) Level 1 comprising 64 sequences of 160 bits (total 10,240 bits) and a threshold rate of 60/64 (**C**) Level 2 comprising 256 sequences of 640 bits (total 163,840 bits) and a threshold rate of 248/256 (**D**) Level 3 comprising 400 sequences of 2,048 bits (total 819,200 bits) and threshold rate of 390/400.

**
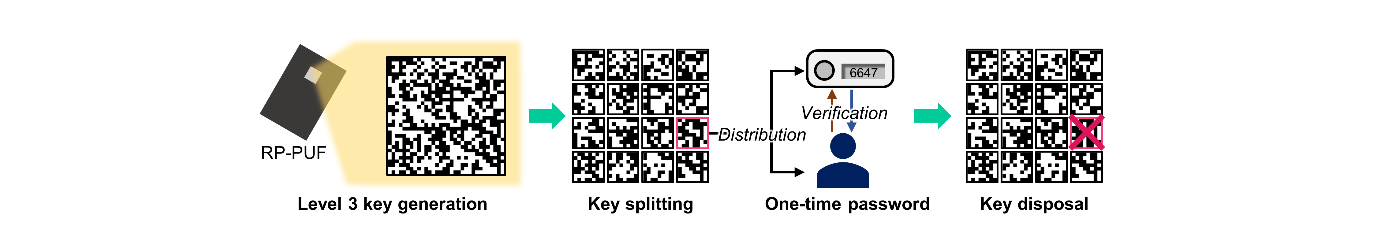
**

**Figure S25. Detailed scenarios for one-time password (OTP) authentication.** Level 3 key is divided into multiple keys for OTP verification of the IoT device. The used key is disposed of after the authentication process; therefore, the divided level 3 key can be regarded as a one-time password.

**
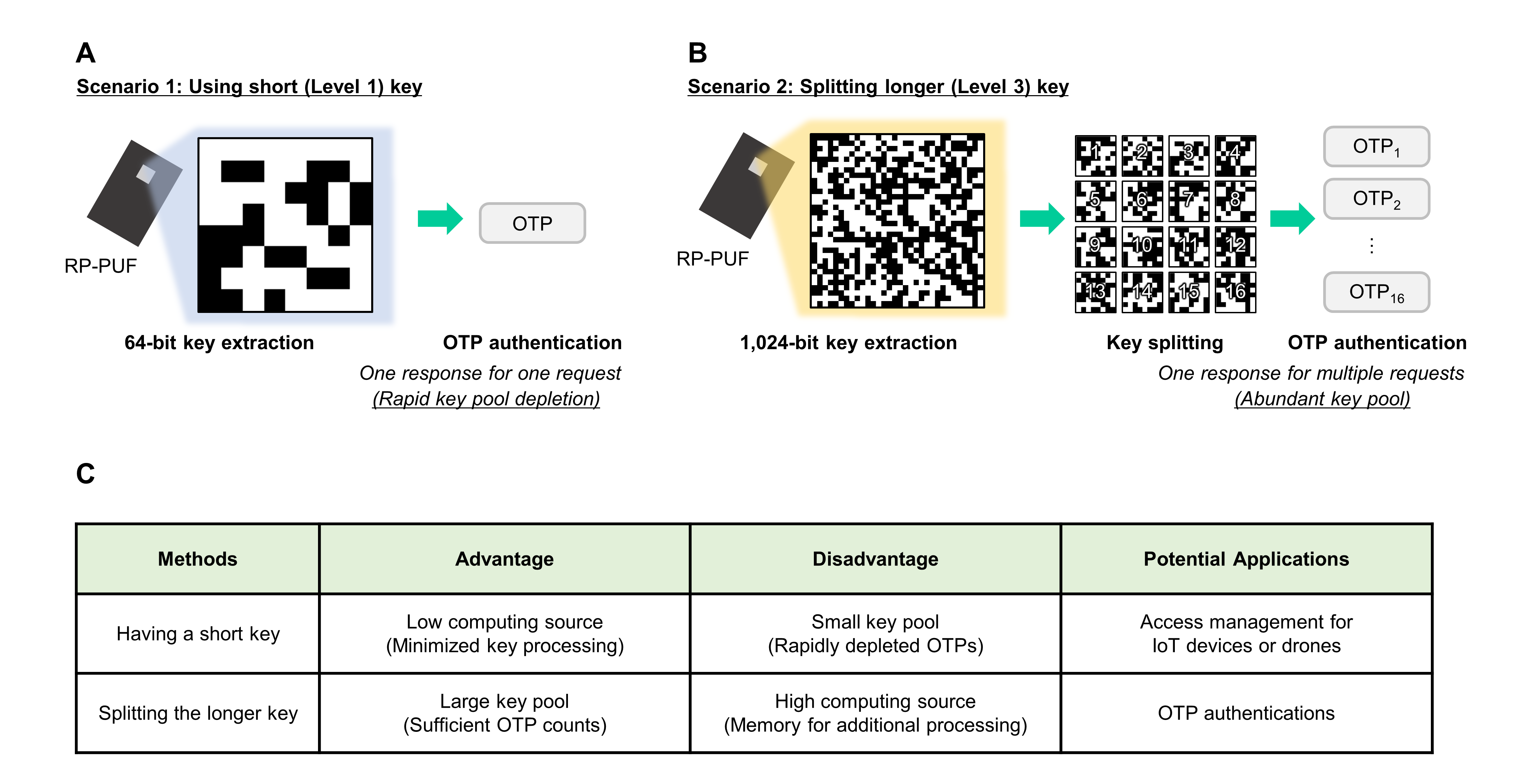
**

**Figure S26. Comparison of two scenarios for extracting level 1 bits and level 3 keys for OTP authentication.** (**A**) Utilization of directly extracting the short key and (**B**) short keys derived by key splitting from a long key. Using the level 1 key for OTP request has the disadvantage of a small key pool, which can be more rapidly depleted than splitting the level 3 key (i.e., 1,024-bit). Therefore, we suggest splitting level 3, providing an abundant key pool in the OTP authentication scenario. (**C**) A summarized table for comparison of two scenarios, having a short key and splitting the longer key. Nevertheless, the level 1 key can be utilized for access management for identification on lightweight devices such as IoT devices or drones, owing to the low memory consumption.


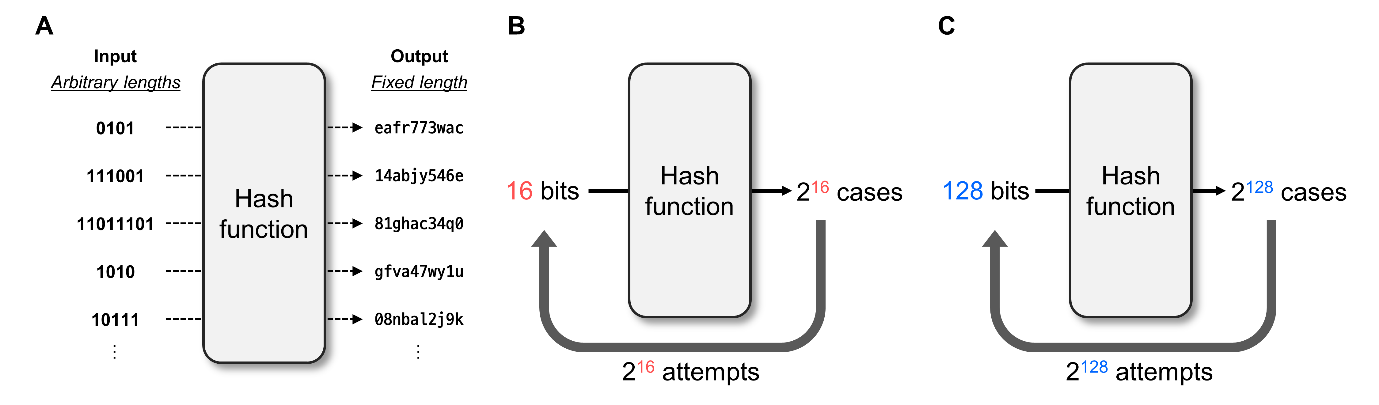


**Figure S27. Hierarchical source for hash functions.** (**A**) The hash function deriving arbitrary bit lengths into a fixed length. (**B**) Short-length input space for small output space and (**C**) long-length input space for large output space. The number of required attempts to find the target hash value increases exponentially with respect to the input length. Combined with hash functions, the key space provides appropriate security strength tailored to the proposed environment, while preventing excessive memory consumption.


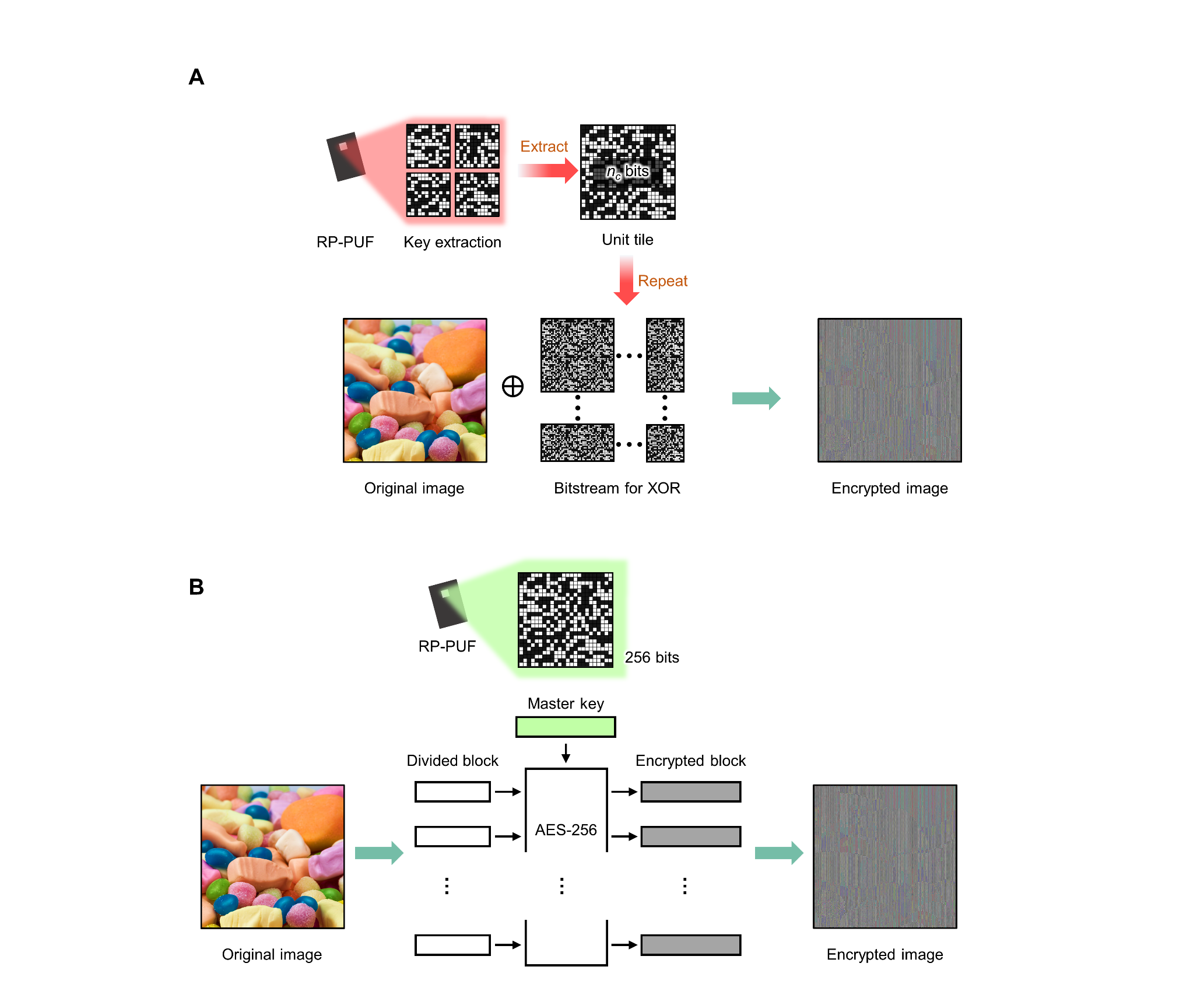


**Figure S28. Schematic of image encryption algorithms for comparison.** (**A**) XOR masking. From extracted keys, a unit with a length of *n_c_* is selected. The unit tile is repeated to match the size of the original image, and the repeated bitstream is operated to the original image using XOR. (**B**) AES-256 ECB mode. In this protocol, all divided blocks are encrypted with the same master key of AES-256.

**
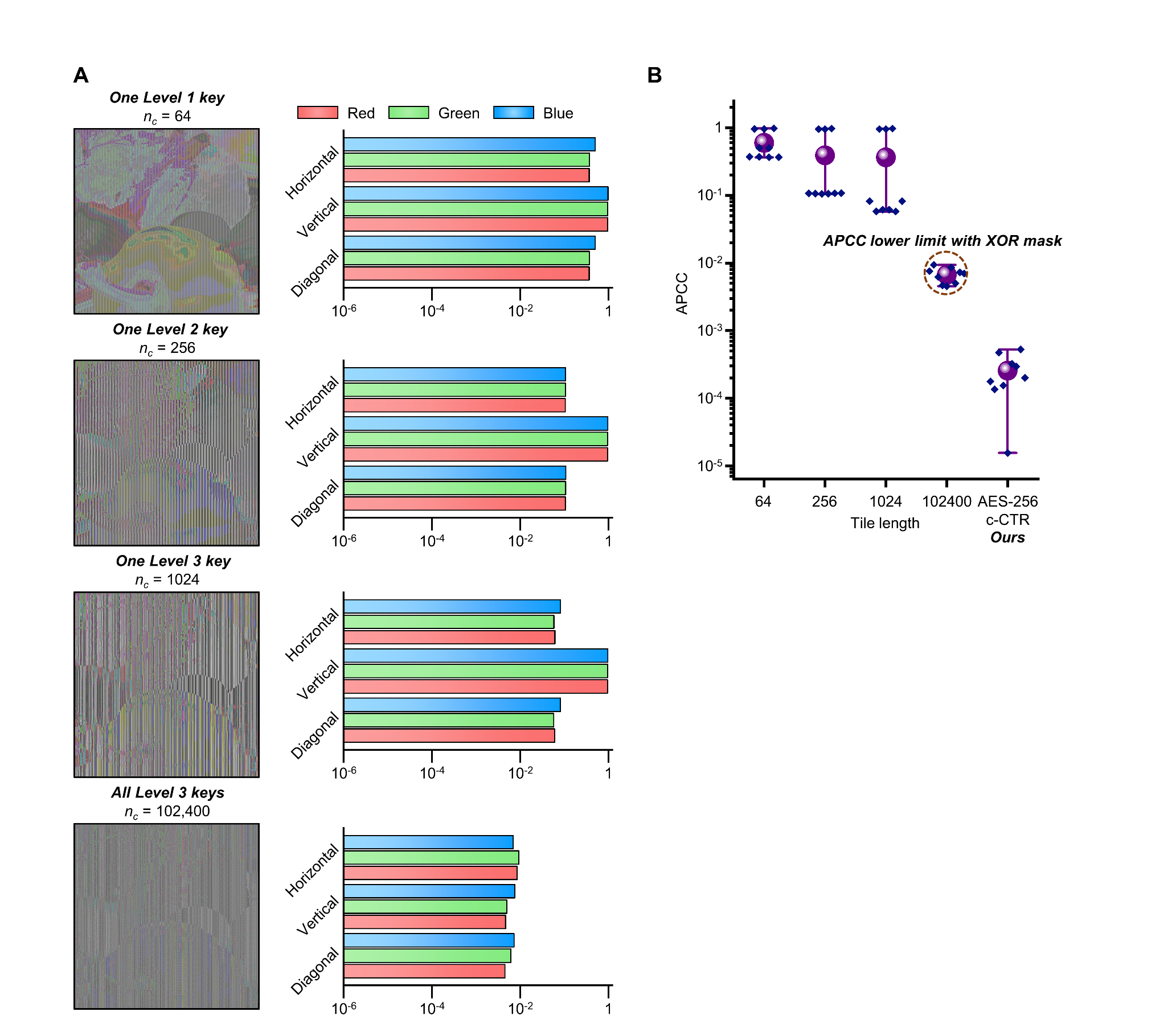
**

**Figure S29. Image encryption with XOR masking.** (**A**) Demonstration results of XOR masking for various *n_c_*. The unit tile length of 64, 256, and 1,024 corresponds to the key length of each level, and 102,400 is derived from the concatenation of the entire level 3 space of RP-PUF. (**B**) Adjacent pixel correlation coefficient (APCC) is measured for the RGB color channel, and three directions (*i.e.*, horizontal, vertical, and diagonal). APCC distribution for unit tile length reveals that mere XOR masking exhibits higher APCC than the proposed AES-256 c-CTR encryption protocol.

**
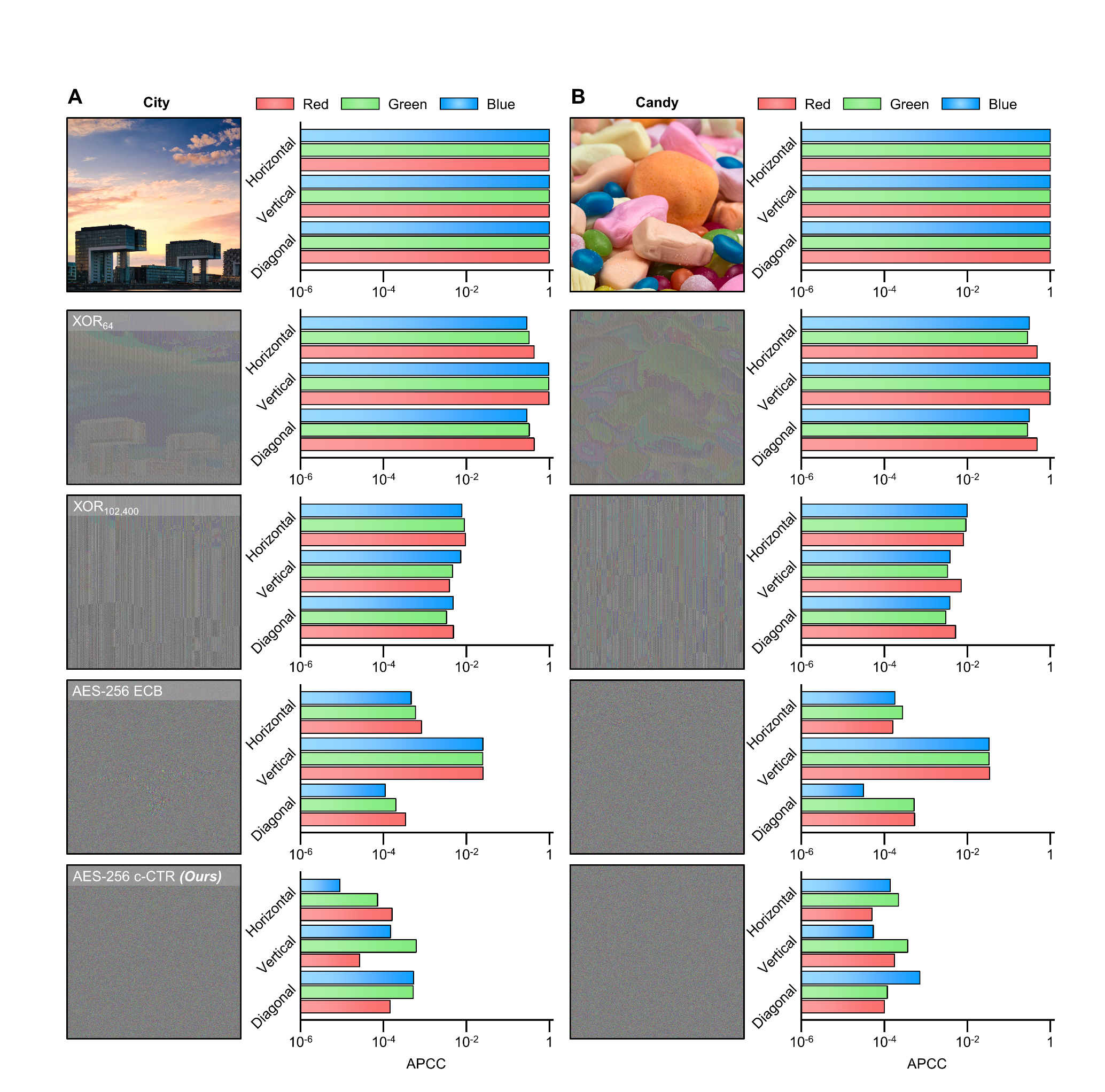
Figure S30. Demonstration results of other images.** Generality of the proposed AES-256 c-CTR mode. The image encryption and APCC measurement across algorithms is performed for different images, (**A**) city and (**B**) candy.


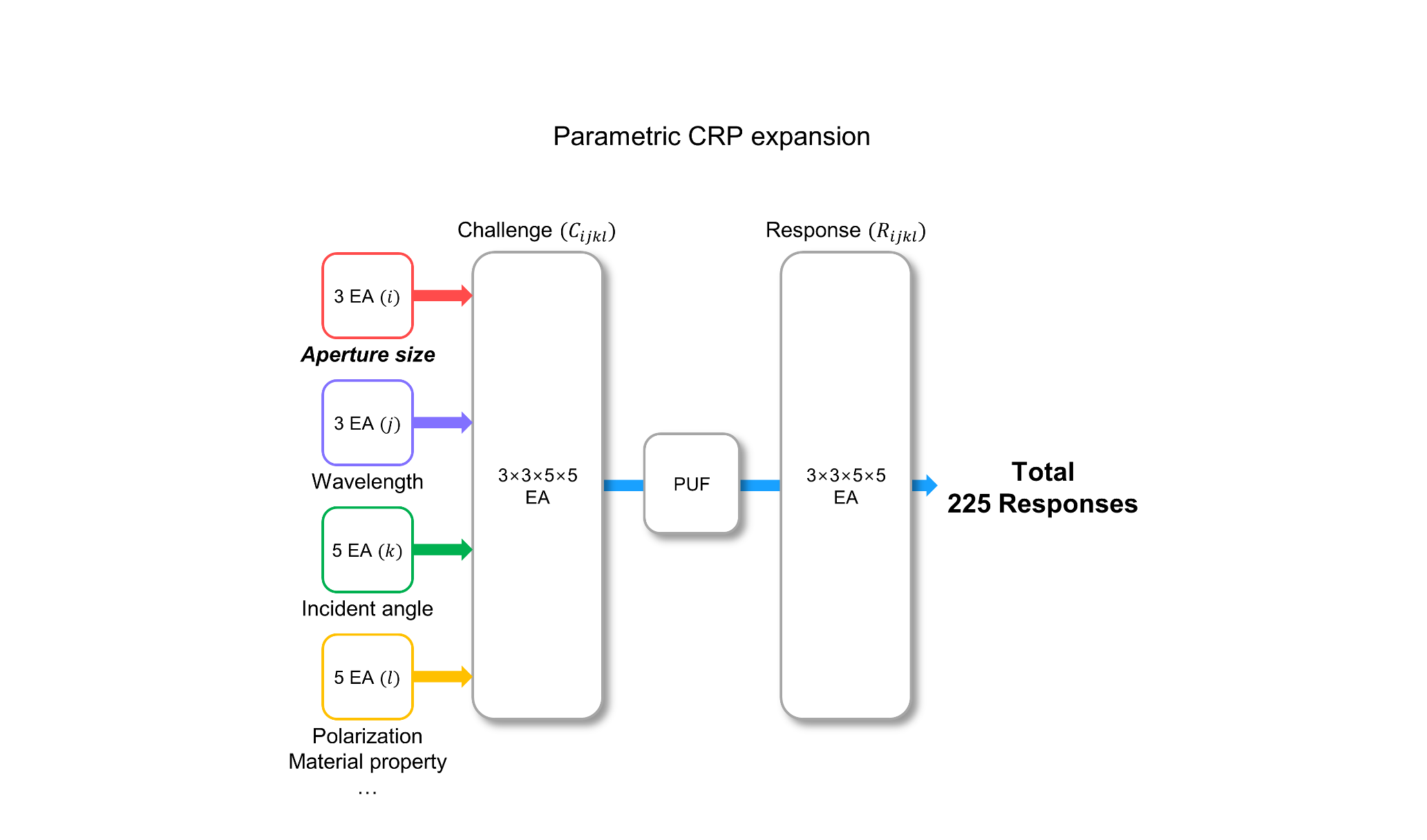


**Figure S31. Parametric expansion of challenge-response pair space. Challenge parameters can independently contribute to and expand the degree of freedom of the challenge space.** Accordingly, the CRP space is expanded multiplicatively by utilizing multiple parameters for the challenge space, leading to an extensive repertoire of encryption keys. In this work, aperture size, wavelength, and incident angle are employed (the available numbers for each parameter were set arbitrarily in the figure). For future work, polarization or adjustable material properties can be considered as other parameters.

**References**

[R1] Pappu, R., Recht, B., Taylor, J., & Gershenfeld, N. (2002). Physical one-way functions. Science, 297(5589), 2026-2030.

[R2] Jing, L., Xie, Q., Li, H., Li, K., Yang, H., Ng, P. L. P., ... & Chen, P. Y. (2020). Multigenerational crumpling of 2D materials for anticounterfeiting patterns with deep learning authentication. Matter, 3(6), 2160-2180.

[R3] Kim, J. H., Jeon, S., In, J. H., Nam, S., Jin, H. M., Han, K. H., ... & Kim, S. O. (2022). Nanoscale physical unclonable function labels based on block copolymer self-assembly. Nature Electronics, 5(7), 433-442.

[R4] Kim, Y., Lim, J., Lim, J. H., Hwang, E., Lee, H., Kim, M., ... & Hong, S. (2023). Reconfigurable multilevel optical PUF by spatiotemporally programmed crystallization of supersaturated solution. Advanced Materials, 35(22), 2212294.

[R5] Nocentini, S., Rührmair, U., Barni, M., Wiersma, D. S., & Riboli, F. (2024). All-optical multilevel physical unclonable functions. Nature materials, 23(3), 369-376.

[R6] Ma, M., Jiang, Z., Ma, T., Gao, X., Li, J., Liu, M., ... & Jiang, X. (2024). Robust PUF Label Authentication System Synergistically Constructed by Hierarchical Pattern of Self‐assembled Phase‐Separation Encrypted Wrinkle and Deep Learning Model. Advanced Functional Materials, 34(44), 2405239.

[R7] Wang, K., Shi, J., Lai, W., He, Q., Xu, J., Ni, Z., ... & Yang, D. (2024). All-silicon multidimensionally-encoded optical physical unclonable functions for integrated circuit anti-counterfeiting. Nature Communications, 15(1), 3203.

[R8] Jiao, F., Lin, C., Dong, L., Mao, X., Wu, Y., Dong, F., ... & Shan, C. X. (2024). Silicon Vacancies Diamond/Silk/PVA Hierarchical Physical Unclonable Functions for Multi‐Level Encryption. Advanced Science, 11(23), 2308337.

[R9] Lin, X., Li, Q., Tang, Y., Chen, Z., Chen, R., Sun, Y., ... & Li, Q. (2024). Physical unclonable functions with hyperspectral imaging system for ultrafast storage and authentication enabled by random structural color domains. Advanced Science, 11(31), 2401983.

[R10] Ahn, J., Park, T., Kang, T., Im, S. G., Seo, H., Kim, B. H., ... & Oh, S. J. (2025). Nanoseed-based physically unclonable function for on-demand encryption. Science Advances, 11(17), eadt7527.
